# Dynamics and regulatory roles of RNA m^6^A methylation in unbalanced genomes

**DOI:** 10.7554/eLife.100144

**Published:** 2025-01-24

**Authors:** Shuai Zhang, Ruixue Wang, Kun Luo, Shipeng Gu, Xinyu Liu, Junhan Wang, Ludan Zhang, Lin Sun

**Affiliations:** 1 Key Laboratory of Cell Proliferation and Regulation Biology of Ministry of Education, College of Life Sciences, Beijing Normal University Beijing China; 2 Beijing Key Laboratory of Gene Resource and Molecular Development, College of Life Sciences, Beijing Normal University Beijing China; University of Colorado School of Medicine United States; National Cancer Institute United States

**Keywords:** aneuploidy, m^6^A, unbalanced genomes, epigenetics, *D. melanogaster*

## Abstract

*N*^6^-methyladenosine (m^6^A) in eukaryotic RNA is an epigenetic modification that is critical for RNA metabolism, gene expression regulation, and the development of organisms. Aberrant expression of m^6^A components appears in a variety of human diseases. RNA m^6^A modification in *Drosophila* has proven to be involved in sex determination regulated by *Sxl* and may affect X chromosome expression through the MSL complex. The dosage-related effects under the condition of genomic imbalance (i.e. aneuploidy) are related to various epigenetic regulatory mechanisms. Here, we investigated the roles of RNA m^6^A modification in unbalanced genomes using aneuploid *Drosophila*. The results showed that the expression of m^6^A components changed significantly under genomic imbalance, and affected the abundance and genome-wide distribution of m^6^A, which may be related to the developmental abnormalities of aneuploids. The relationships between methylation status and classical dosage effect, dosage compensation, and inverse dosage effect were also studied. In addition, we demonstrated that RNA m^6^A methylation may affect dosage-dependent gene regulation through dosage-sensitive modifiers, alternative splicing, the MSL complex, and other processes. More interestingly, there seems to be a close relationship between MSL complex and RNA m^6^A modification. It is found that ectopically overexpressed MSL complex, especially the levels of H4K16Ac through MOF, could influence the expression levels of m^6^A modification and genomic imbalance may be involved in this interaction. We found that m^6^A could affect the levels of H4K16Ac through MOF, a component of the MSL complex, and that genomic imbalance may be involved in this interaction. Altogether, our work reveals the dynamic and regulatory role of RNA m^6^A modification in unbalanced genomes, and may shed new light on the mechanisms of aneuploidy-related developmental abnormalities and diseases.

## Introduction

Epigenetic modifications regulate gene expression in response to environmental changes and play important roles in the development of organisms and a variety of human diseases (Jaenisch and Bird, 2003; Lence et al., 2017). In addition to DNA and chromatin modifications, which are well studied, more than 100 RNA chemical modifications have been identified in cells to date (Lee et al., 2014; Roundtree et al., 2017a). As a marker of post-transcriptional regulation, RNA modifications participate in almost all aspects of RNA metabolism (Roundtree et al., 2017a). *N*^6^-methyladenosine (m^6^A) is the most prevalent internal modification in many eukaryotic messenger RNAs (mRNAs) and long noncoding RNAs (lncRNAs) (Lee et al., 2014; Roundtree et al., 2017a; Yang et al., 2018), which widely affects RNA alternative splicing (Dominissini et al., 2012), export (Roundtree et al., 2017b), stability (Wang et al., 2014b), and translation (Meyer et al., 2015). RNA m^6^A modifications have been found to be enriched on the transcripts of genes that regulate development and cell fate specification (Dominissini et al., 2012; Meyer et al., 2012; Geula et al., 2015), and some m^6^A sites are regulated in a tissue- or disease-specific manner (Zaccara et al., 2019). In addition, the abnormal expression of m^6^A components is related to the tumorigenesis, proliferation, and metastasis of many types of cancers (Pinello et al., 2018; Ma et al., 2019). Therefore, it is of great significance to study RNA m^6^A methylation for revealing the mechanisms of gene expression regulation and human diseases.

At present, there are few studies on RNA m^6^A modification in *Drosophila*, possibly due to its relatively low abundance (m^6^A/A<0.2%) (Haussmann et al., 2016), and the mutation of some m^6^A component genes will affect their viability and fertility (Hongay and Orr-Weaver, 2011; Haussmann et al., 2016; Kan et al., 2017). However, as a model organism with specific genetic and developmental advantages, *Drosophila* remains an excellent tool for studying the roles of epigenetic modifications in gene regulation, individual development, and disease process. Several components of the *Drosophila* m^6^A methyltransferase complex (*Ime4*, *dMettl14*, and *fl(2)d* constitute the core complex, with *vir* and *nito* acting as cofactors) and an m^6^A reader protein (*Ythdc1*) have been identified, all of which have homologues in mammals (Lence et al., 2017). Deletion of the major methyltransferase gene *Ime4* or the reader *Ythdc1* causes locomotion defects, and the splicing of sex-determining factor *Sxl* is affected (Haussmann et al., 2016; Lence et al., 2016; Kan et al., 2017). However, homozygous mutations in *fl(2)d*, *vir*, and *nito* were lethal, suggesting that these subunits have important functions other than methylation (Penn et al., 2008; Yan and Perrimon, 2015; Kan et al., 2017; Lence et al., 2017). RNA m^6^A modification has an obvious sexual dimorphism in *Drosophila*, and reduced m^6^A levels severely decreased the survival of females. It is thought to be due to the derepression of *msl-2* caused by aberrantly spliced *Sxl*, which forms the male-specific lethal (MSL) complex that associates with the X chromosome (Haussmann et al., 2016).

Dosage compensation is a widespread phenomenon in unbalanced genomes (Lucchesi, 2018; Birchler and Veitia, 2021). The deletion or duplication of some chromosomes rather than the whole chromosome set leads to genomic imbalance, i.e., aneuploidy (Orr et al., 2015). Aneuploid variation is usually detrimental to organisms (Birchler and Veitia, 2012; Orr et al., 2015; Birchler and Veitia, 2021), and is associated with developmental abnormalities, mental retardation, and various congenital defects (Williams et al., 2008; Huang et al., 2021; Sanchez-Pavon et al., 2021), possibly due to disorders in their gene expression systems (Prestel et al., 2010; Letourneau et al., 2014). Studies across species have pointed out that there is genome-wide *trans* modulation in aneuploidy, and the genes on the varied chromosomes are compensated to a certain extent, while genes located on the rest of the genome are mainly regulated in the opposite direction to the changes of chromosome numbers, which is known as the inverse dosage effect (Birchler and Veitia, 2012; Birchler and Veitia, 2021; Sun et al., 2013c; Hou et al., 2018; Shi et al., 2021). Histone modification (Zhang et al., 2021a), chromatin remodeling (Birchler, 2016), lncRNAs (Zhang et al., 2023), and microRNAs Shi et al., 2022 have all been shown to play a role in genomic imbalance.

Because of the haploinsufficiency for X-linked genes, heterogametic individuals in organisms with XY sex determination systems could be regarded as analogous to aneuploidy (Disteche, 2016), including humans and *Drosophila*. Some studies have linked histone H4 lysine 16 acetylation (H4K16Ac) and non-coding *roX* RNAs to the dosage compensation of *Drosophila* (Park et al., 2010; Conrad et al., 2012). On the other hand, the compensation of X chromosome in human is thought to be regulated by lncRNA X-inactive specific transcript (*XIST*) (Jordan et al., 2019). Interestingly, in *Drosophila*, RNA m^6^A modification indirectly affects gene expression through *Sxl* and *msl-2*; while in human, m^6^A methylation of the key lncRNA *XIST* is necessary for the silencing of gene transcription on one of the female X chromosomes (Patil et al., 2016). Moreover, most tumor cells have genomic instability and high levels of aneuploidy (Ben-David and Amon, 2020; Chiarle, 2021), and at the same time, m^6^A component genes are often aberrantly expressed in various cancers (Pinello et al., 2018; Ma et al., 2019).

An increasing number of studies have found that dosage-related effects in aneuploidy may be the integration of multiple modulations rather than through a single mechanism (Prestel et al., 2010; Birchler, 2016). The effects of genomic imbalance are complicated, and the model of dosage compensation and global gene regulation in *Drosophila* has been extended to autosomal aneuploidies and sex chromosome aneuploid metafemales where MSL complexes are not assembled (Sun et al., 2013b; Sun et al., 2013c; Zhang et al., 2021b). In addition, genomic imbalance and *trans* regulatory mechanisms in other species such as maize, *Arabidopsis*, and humans have also been investigated (Hou et al., 2018; Raznahan et al., 2018; Shi et al., 2021; Yang et al., 2021; San Roman et al., 2023). To reveal the role of RNA m^6^A modification in unbalanced genomes, we studied the dynamic changes and regulatory functions of m^6^A methylation under genetic imbalance conditions using autosomal and sex chromosome aneuploid *Drosophila* maintained in our laboratory. Meanwhile, dosage-sensitive modifiers, differential alternative splicing events, the MSL complex, and other factors that may mediate the relationships between RNA m^6^A modification and the dosage-related effects of aneuploidy were also investigated. In summary, we provided a comprehensive picture of RNA m^6^A methylation in unbalanced genomes.

## Results

### The responses of m^6^A components under genomic imbalance

**Figure 1.**
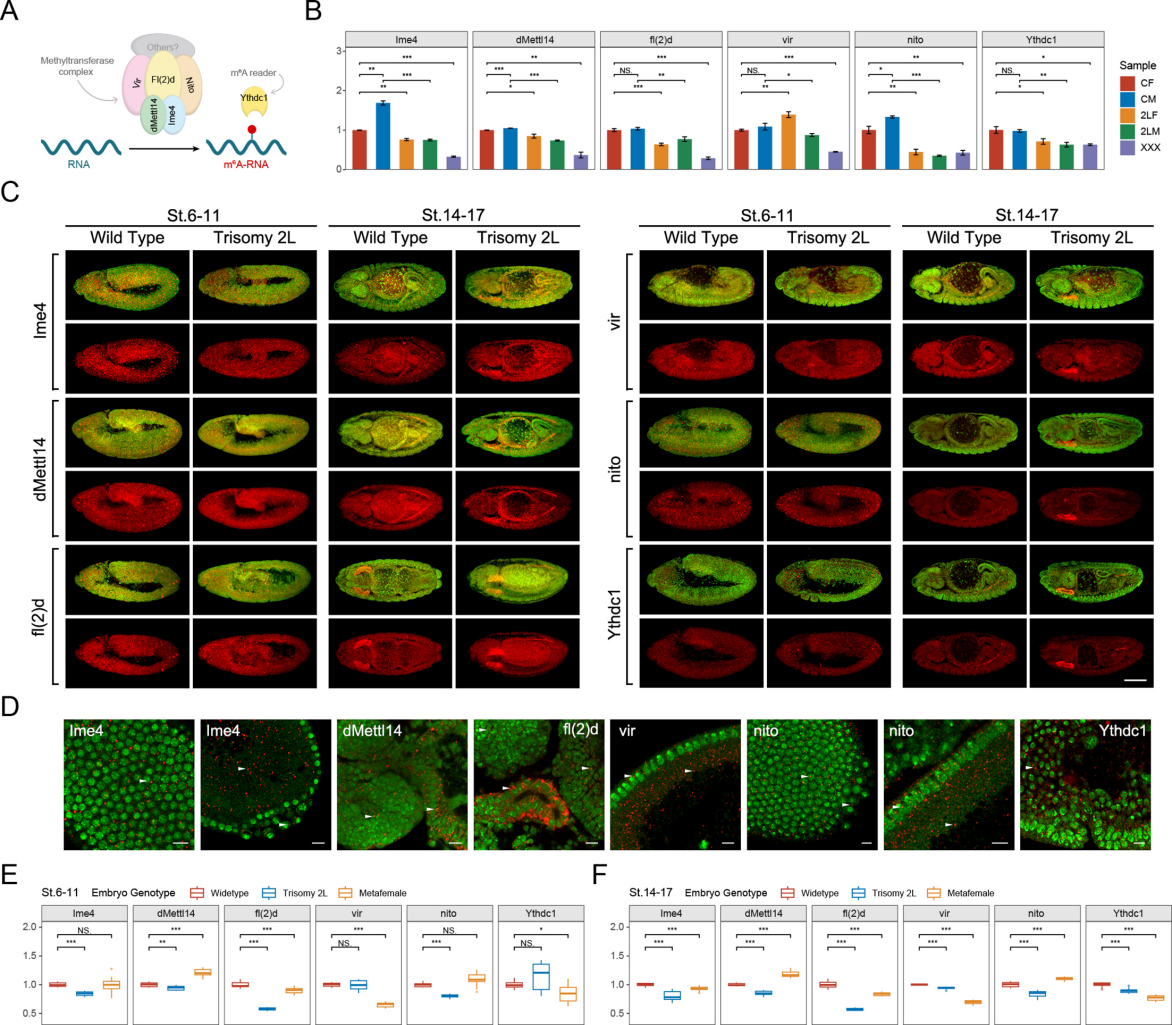
The responses of m^6^A methyltransferases and reader protein under the condition of genomic imbalance. (**A**) Schematic diagram of m^6^A components in *Drosophila*. (**B**) RT-qPCR analysis of messenger RNA (mRNA) levels of m^6^A methyltransferases and reader protein in third instar larvae of wildtype and trisomy *Drosophila*. CF, wildtype female control; CM, wildtype male control; 2LF, trisomy 2L female; 2LM, trisomy 2L male; XXX, metafemale; 2L, chromosome 2 left arm. Sample size = 3. Student’s *t* test *p<0.05, **p<0.01, ***p<0.001. (**C**) Subembryonic distribution patterns of the transcripts of m^6^A components in wildtype and trisomy 2L *Drosophila*. The names of the genes were shown in the left of the pictures; the genotypes and stages were shown above. Red, probes; green, DAPI. Scale bar, 100 μm. (**D**) Subcellular localization of probe signals. Probe name was written in the corner of each picture. Red, probe; green, DAPI. Arrowheads indicate the foci of probe signals. The tissue types are (1) blastoderm nuclei; (2) yolk plasm and pole cells; (3) brain and midgut; (4) salivary gland and midgut; (5) blastoderm nuclei and yolk cortex; (6) blastoderm nuclei and pole cells; (7) blastoderm nuclei and yolk cortex; (8) germ band. Scale bars, 10 μm. (**E,F**) The expression levels of m^6^A component genes in stage 6–11 (**E**) and stage 14–17 (**F**) represented by relative fluorescence intensity of probes compared with DAPI signals. The expression of wildtype embryos was set as one. Sample size = 10. Student’s *t* test *p<0.05, **p<0.01, ***p<0.001.

RNA m^6^A methylation is a reversible epigenetic modification, and its dynamic process is mediated by m^6^A methyltransferases (writers), demethylases (erasers), and m^6^A recognition proteins (readers) (Lee et al., 2014; Yang et al., 2018; Zaccara et al., 2019; Figure 1A). We first detected the expression of m^6^A components in *Drosophila* larvae with karyotypes of normal diploids and trisomies that have an additional chromosome arm using RT-qPCR to determine whether RNA m^6^A dynamics are affected by correlative enzymes in unbalanced genomes (Figure 1B; Figure 1—figure supplement 1A and B). It was found that the transcription levels of most m^6^A writers and the major m^6^A reader are down-regulated in aneuploids compared with their respective sex-corresponding controls (Figure 1B). Unlike the other components, the cofactor *vir* of methyltransferase complex is up-regulated in trisomy 2L females (Haussmann et al., 2016; Lence et al., 2016; Kan et al., 2017). Because RNA m^6^A modification is enriched in the nervous system of *Drosophila*, the brains of aneuploid third instar larvae were also used to detect the expression of m^6^A components. As expected, a decreased expression trend of m^6^A components similar to that of the whole larvae was observed (Figure 1—figure supplement 1C).

**Figure 1—figure supplement 1.**
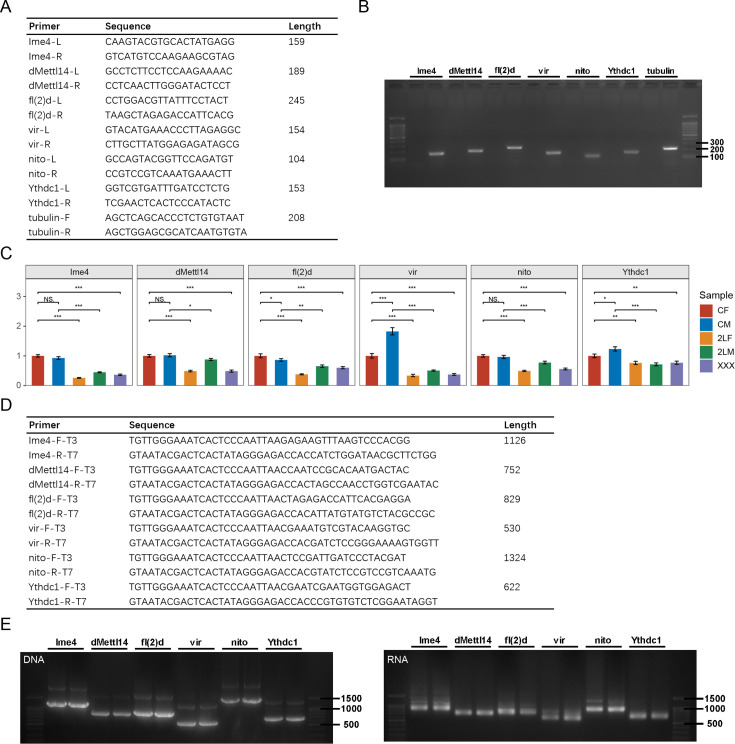
Primers of m^6^A methyltransferases and reader protein for RT-qPCR and TSA-FISH. (**A**) The sequences of RT-qPCR primers. (**B**) Agarose gel electrophoresis validation of qPCR primers. The left lane of each gene is negative control with primers only and no DNA substrate. (**C**) RT-qPCR analysis of messenger RNA (mRNA) levels of m^6^A methyltransferases and reader protein in the brains of wildtype and trisomy *Drosophila* larvae. CF, wildtype female control; CM, wildtype male control; 2LF, trisomy 2L female; 2LM, trisomy 2L male; XXX, metafemale. Sample size = 3. Student’s *t* test *p<0.05, **p<0.01, ***p<0.001. (**D**) The sequences of FISH primers. (**E**) The sizes of the products of the FISH primers after PCR amplification and in vitro transcription were tested by agarose gel electrophoresis. 2L, chromosome 2 left arm; RT-qPCR, real-time quantitative PCR; TSA-FISH, tyramide signal amplification-based fluorescence in situ hybridization.

**Figure 1—figure supplement 2.**
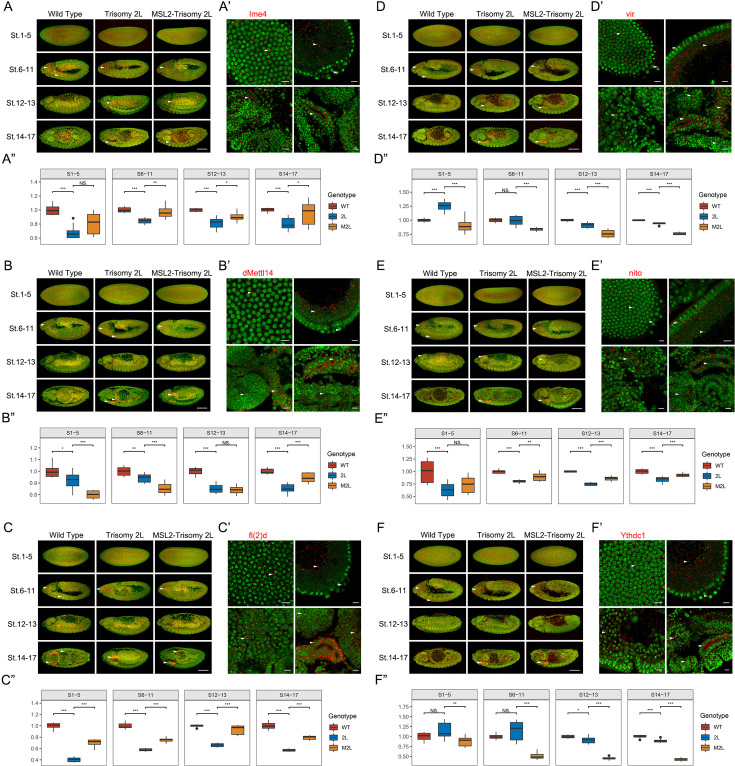
Embryo tyramide signal amplification-based fluorescence in situ hybridization (TSA-FISH) of m^6^A components in wildtype, trisomy, and MSL2-overexpressed trisomy *Drosophila*. The images represent m^6^A component *Ime4* (**A–A”**), *dMettl14* (**B–B”**), *fl(2)d* (**C–C”**), *vir* (**D–D”**), *nito* (**E–E”**), and *Ythdc1* (**F–F”**), respectively. (**A–F**) Gene expression patterns in entire embryos. The genotypes of the samples were shown above, and the development stages (St.) were shown in the left of the pictures. The red pseudocolor represents signal from the probes, and green is the signal of nucleus. Arrowheads indicate regions where the probe signal are enriched. Scale bars, 100 μm. (**A’–F’**) RNA subcellular location patterns of m^6^A components. Arrowheads indicate probe signals. Scale bars, 10 μm. (**A”–F”**) The expression levels of m^6^A component genes represented as relative fluorescence intensity of the probes compared with DAPI. The expression of wildtype embryos was set as base line. WT, wildtype; 2L, trisomy 2L; M2L, MSL2-overexpressed trisomy 2L. Sample size = 10. Student’s *t* test *p<0.05, **p<0.01, ***p<0.001.

Previous studies have pointed out that the m^6^A methylomes have temporal and spatial specificity in the development process of organisms, especially for embryonic development and cell differentiation (Meyer and Jaffrey, 2014; Wang et al., 2014a). Aberrant expression of m^6^A components may cause defects in embryogenesis and even early embryonic lethality (Zhong et al., 2008; Wang et al., 2014a; Geula et al., 2015; Zhao et al., 2017). Therefore, we designed probes of m^6^A components (Figure 1—figure supplement 1D and E) to examine the mRNA expression and localization patterns during early embryonic development of aneuploid *Drosophila* using high-resolution tyramide signal amplification-based fluorescence in situ hybridization (TSA-FISH) (Lécuyer et al., 2007; Jandura et al., 2017). The results showed that the subembryonic distribution patterns of the five components of m^6^A methyltransferase complex and one m^6^A reading protein are similar in wildtype and aneuploidies (Figure 1C and D; Figure 1—figure supplement 2A–F). At the blastoderm stage (stage 1–5), the probe signals of m^6^A components are widely distributed in the surface cell layer, yolk plasma, yolk cortex, and show a pattern of basal enrichment (Figure 1—figure supplement 2A–F). For the gastrulae at stage 6–11, the transcripts of m^6^A components are mainly located in head, amnioproctodeal invagination, germ band, and as in previous studies (Lence et al., 2016), and show an enrichment in neuroectoderm (Figure 1C; Figure 1—figure supplement 2A–F). At later stages of embryonic development (stages 12–13 and 14–17), the probes are distributed in brain, ventral nerve cord, midgut, and salivary gland (Figure 1C; Figure 1—figure supplement 2A–F).

By observing the subcellular localization of the probe signals of m^6^A components at higher magnification, it can be found that most of the mRNAs of these genes have nuclei-associated localization patterns (Figure 1D; Figure 1—figure supplement 2A’–F’). In the early and late stages of embryonic development, the probe signals of *Ime4*, *dMettl14*, *fl(2)d*, *vir*, and *Ythdc1* form dense small foci near the nucleus, which is a perinuclear distribution (Figure 1D; Figure 1—figure supplement 2A’–F’). For *nito*, in addition to the perinuclear signals, there is also an obvious signal of intranuclear localization during early embryogenesis, which is manifested as one or two small foci in the blastoderm nucleus (Figure 1D; Figure 1—figure supplement 2E’).

Although there seems to be no difference in the localization of the transcripts of m^6^A components that we detected in aneuploidy and wildtype embryos, the expression levels of these genes, as determined by relative fluorescence intensity, are significantly changed (Figure 1E and F; Figure 1—figure supplement 2A”–F”). Except for the irregular fluctuations at early embryonic stages, the expression levels of most m^6^A components in aneuploids are lower than those in wildtype at more mature stages (Figure 1E and F; Figure 1—figure supplement 2A”–F”), which is similar to the trend we detected in third instar larvae (Figure 1B; Figure 1—figure supplement 1C). The transcripts of m^6^A methyltransferase and reader protein have specific subembryonic and subcellular localization patterns in the development of *Drosophila* embryos, which may be a mechanism to regulate cellular functions, and ensure appropriate cell growth and differentiation (Lécuyer et al., 2007).

### Genome-wide mapping of RNA m^6^A methylation in aneuploid *Drosophila*

**Figure 2.**
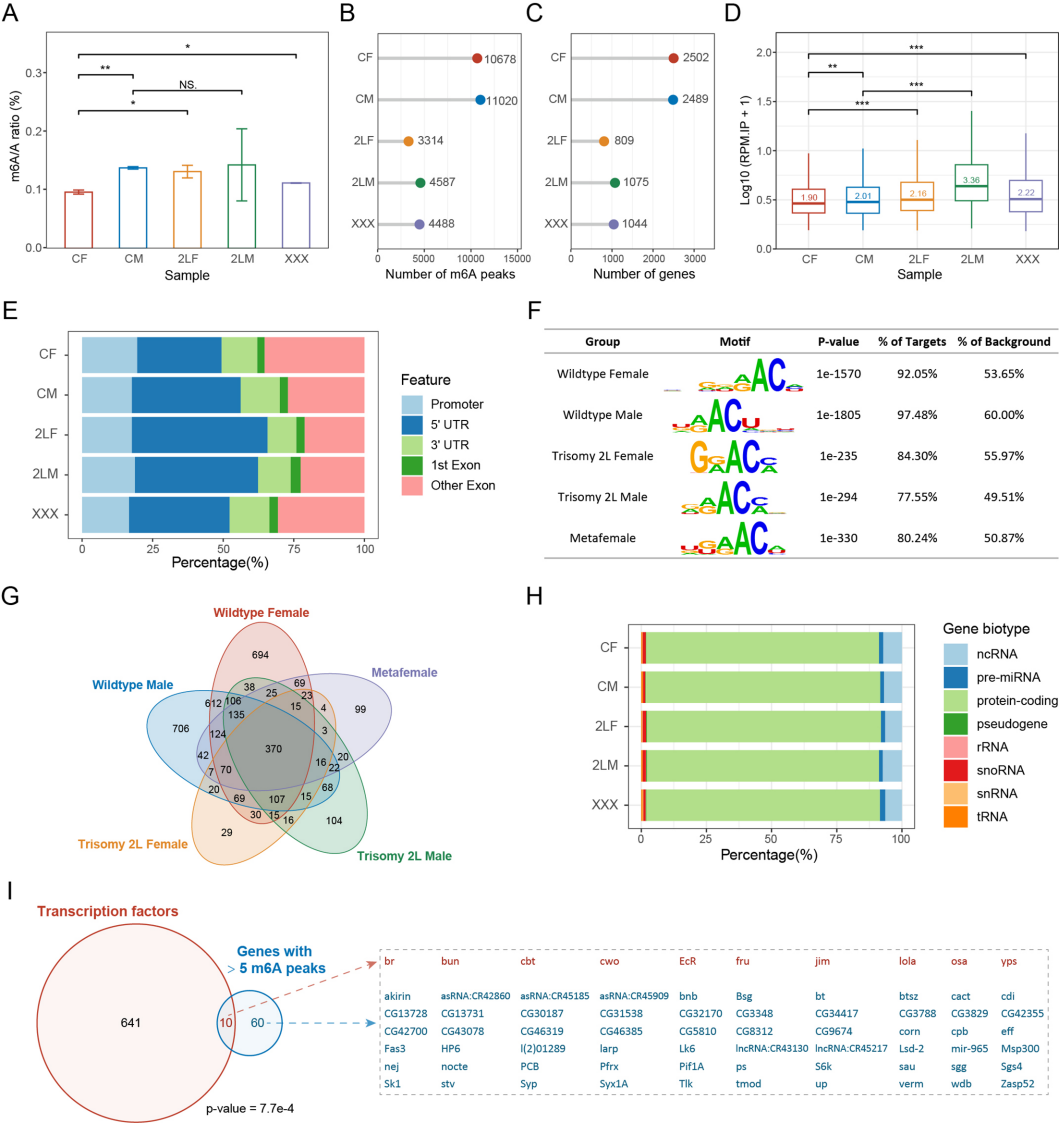
Overview of RNA m^6^A methylation in aneuploid *Drosophila*. (**A**) Global m^6^A abundance in third instar larvae of wildtype and aneuploid *Drosophila*. Data represent the mean of two independent experiments, each containing three or four biological replicates. Quantification was performed using EpiQuik m^6^A RNA Methylation Quantification Kit. Error bar indicates the standard error of the means (SEM). Student’s *t* test *p<0.05, **p<0.01. (**B**) The number of m^6^A peaks identified by MeRIP-Seq. (**C**) The number of m^6^A-modified genes obtained by annotating the peaks. (**D**) Expression levels of m^6^A modification sites in IP samples expressed as log10-transformed reads per million (RPM). The number on the boxplot indicates the median RPM of each sample. Mann-Whitney U test *p<0.05, **p<0.01, ***p<0.001. (**E**) Percentages of peaks localized on different gene features. (**F**) The most enriched motifs obtained by de novo motif analysis of the m^6^A peaks. (**G**) Venn diagram showing the intersection of m^6^A-modified genes in each sample. (**H**) Gene biotypes of m^6^A-modified genes. (**I**) Venn diagram showing the intersection of transcription factors and m^6^A-modified genes with more than five m^6^A peaks in all samples. Seventy genes with more than five m^6^A peaks were listed on the right, with transcription factors in red and others in blue. p-Value indicates one-tailed Fisher’s exact test. CF, wildtype female control; CM, wildtype male control; 2LF, trisomy 2L female; 2LM, trisomy 2L male; XXX, metafemale; 5′UTR, 5′ untranslated region; 3′UTR, 3′ untranslated region; MeRIP-Seq, m^6^A methylated RNA immunoprecipitation sequencing.

Subsequently, we detected the global levels of RNA m^6^A methylation in wildtype and aneuploid *Drosophila* larvae to determine whether m^6^A abundance is altered under the condition of genomic imbalance due to modulation by transmethylases. The overall abundance of m^6^A was represented by the m^6^A/A ratio in total RNA (Figure 2A). It was found that the m^6^A abundance of wildtype males is higher than that of females, but both are lower than 0.2%, which is consistent with the finding that m^6^A levels in *Drosophila* are relatively low (Haussmann et al., 2016; Lence et al., 2016). Females with triple chromosome 2 left arms (2L) and metafemales with triple X chromosomes have significantly higher m^6^A abundances than diploid females, whereas there is no significant difference between trisomy 2L males and wildtype males (Figure 2A). Therefore, genomic imbalance can affect the m^6^A methylation status to some extent, and this epigenetic modification is different between males and females. However, the m^6^A abundance in aneuploidies did not follow the expression levels of transmethylases.

**Figure 2—figure supplement 1.**
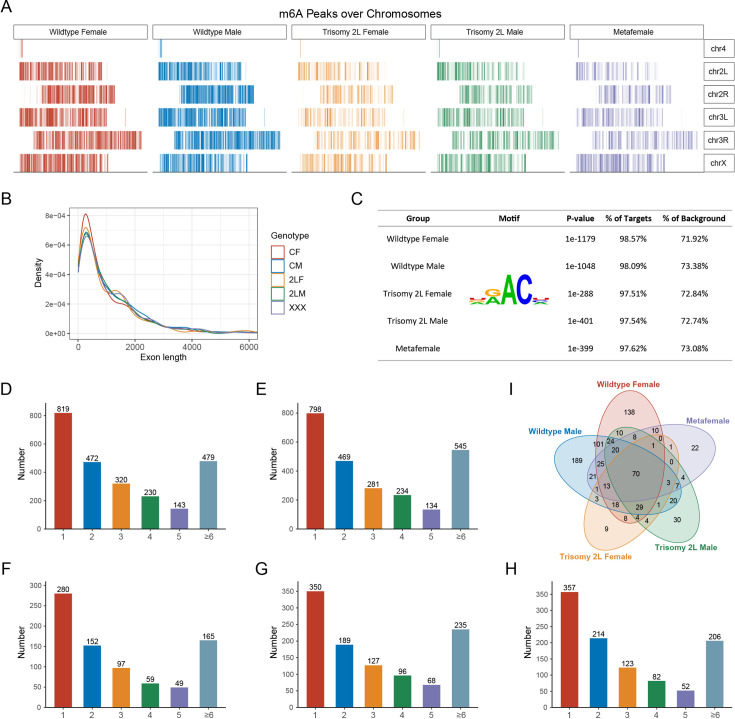
Characteristics of m^6^A-modified genes in wildtype and aneuploid *Drosophila*. (**A**) Distribution of m^6^A peaks along each chromosome. (**B**) Kernel density plot showing the distribution of exon lengths of m^6^A-modified genes. CF, wildtype female control; CM, wildtype male control; 2LF, trisomy 2L female; 2LM, trisomy 2L male; XXX, metafemale. (**C**) Enrichment analysis performed on the known m^6^A consensus motif DRACH (D=G/A/U, R=G/A, H=U/A/C). (**D–H**) Statistics of the number of m^6^A peaks on m^6^A-modified genes in wildtype females (**D**), wildtype males (**E**), trisomy 2L females (**F**), trisomy 2L males (**G**), and metafemales (**H**). (**I**) Venn diagram showing the number of m^6^A-modified genes with more than five m^6^A peaks.

**Figure 2—figure supplement 2.**
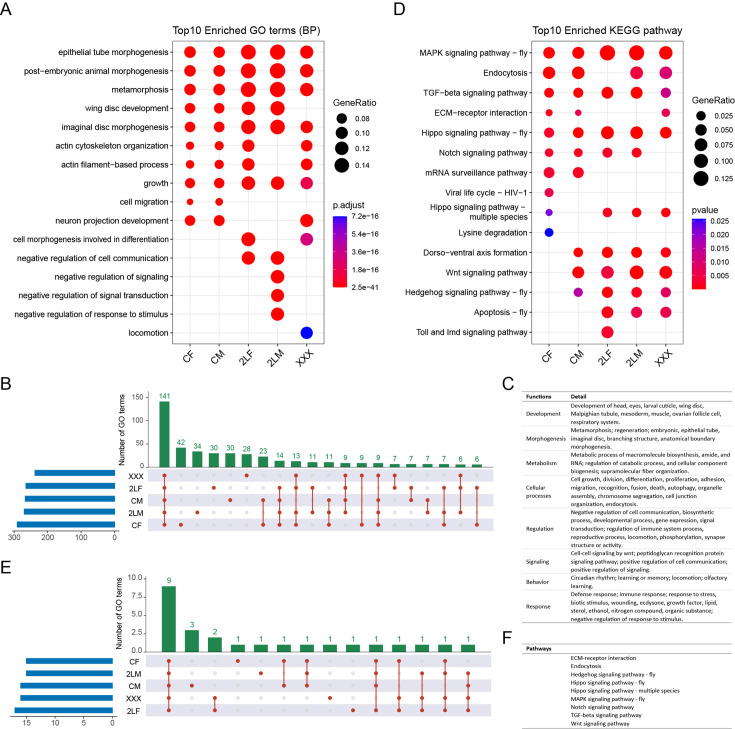
Functional and pathway analysis of m^6^A-modified genes in wildtype and aneuploid *Drosophila*. (**A**) Functional enrichment analysis of m^6^A-modified genes. Top 10 enriched GO terms (Biological Process) with adjusted p-value<0.05 in each sample were shown. (**B**) UpSet plot showing the shared sets of enriched GO terms of m^6^A-modified genes in different groups. (**C**) Summary of the common functions enriched by m^6^A-modified genes in all genotypes. (**D**) KEGG pathway enrichment analysis of m^6^A-modified genes. Top 10 enriched pathways with p-value<0.05 in each sample were shown. (**E**) UpSet plot showing the shared sets of enriched KEGG pathways of m^6^A-modified genes in different groups. (**F**) The pathways enriched in all genotypes were listed. CF, wildtype female control; CM, wildtype male control; 2LF, trisomy 2L female; 2LM, trisomy 2L male; XXX, metafemale; GO, Gene Ontology; BP, Biological Process; KEGG, Kyoto Encyclopedia of Genes and Genomes.

To obtain m^6^A mapping across the transcriptome of aneuploid *Drosophila*, whole larvae with different karyotypes were used for m^6^A methylated RNA immunoprecipitation sequencing (MeRIP-Seq). By identifying consistent peaks in two biological replicates, approximately 10,000 m^6^A peaks were found in wildtype females and males, whereas there were less than half the number of methylation sites in the three kinds of aneuploidies (Figure 2B). When these m^6^A sites were annotated to the genes, the changes in the number of m^6^A-marked genes in samples of different genotypes were in accordance with that of m^6^A peaks, in which there are about 2500 m^6^A-marked genes in wildtype and about 1000 m^6^A-modified genes in trisomies (Figure 2C). Both the number of m^6^A peaks and the number of m^6^A-modified genes are the least in trisomy 2L females (Figure 2B and C). The changes in the number of RNA methylation sites in aneuploids did not coincide with the changes of overall m^6^A abundance, but instead matched the expression of m^6^A components. We speculate that this may be caused by the nonuniformity and heterogeneity of RNA m^6^A modification, including the tissue specificity, the developmental specificity, the different numbers of m^6^A sites in one transcript, the different proportions of methylated transcripts, etc. (Meyer et al., 2012; Meyer and Jaffrey, 2014; Zaccara et al., 2019). Counting the number of reads that mapped to m^6^A peaks in wildtype and aneuploidy MeRIP samples showed that the read levels at m^6^A sites in aneuploidies are significantly higher than that in wildtypes (Mann-Whitney U test p-values<0.001; Figure 2D); further analysis of the relative IP/input ratios showed that the values of aneuploidies are still higher than wildtypes (Mann-Whitney U test p-values<0.001), which confirmed our hypothesis. These results suggest that RNA m^6^A modification exhibits greater heterogeneity in unbalanced genomes.

We next studied the overall characteristics of m^6^A methylation in aneuploid and control *Drosophila*. m^6^A peaks are localized along all autosomes and sex chromosomes, and the numbers of peaks are correlated with chromosome length (Figure 2—figure supplement 1A). Methylation sites are widely distributed on 5’UTR (30–50%), 3’UTR (10–15%), promoter (15–20%), and internal exons (20–35%), and the aneuploidies appeared to have a higher proportion of 5’UTR peaks and a lower proportion of exon peaks than the wildtype (Figure 2E). The highest values of density distributions of the length of exons containing m^6^A peaks are around 250 bp, and approximately 90% of the exons are longer than the typical 140 bp (Figure 2—figure supplement 1B). De novo motif analysis of m^6^A methylation peaks in each sample showed that the top-ranked motif is consistent with the conserved m^6^A motif DRACH (D=G/A/U, R=G/A, H=U/A/C; Figure 2F). In addition, enrichment analysis of the known motif DRACH showed that it is significantly enriched in all samples, and more than 97% of the m^6^A sites have this sequence (Figure 2—figure supplement 1C).

There are 370 common genes to which the m^6^A peaks were annotated in all genotypes (Figure 2G). We classified the m^6^A-modified genes in each sample and found that they were mostly protein-coding genes, whereas only about 7% were ncRNAs (Figure 2H). As described in previous studies (Meyer et al., 2012; Zaccara et al., 2019), most methylated RNAs have one m^6^A peak (Figure 2—figure supplement 1D–H). However, there are still some genes whose transcripts can be highly methylated and contain more than five m^6^A sites (Figure 2—figure supplement 1D–I). It was found that transcription factors were significantly enriched in 70 genes with more than five m^6^A peaks in both wildtype and aneuploidy (Fisher’s exact test p-value = 7.7e-4; Figure 2I; Figure 2—figure supplement 1I). Thus, specific types of genes may be preferentially targeted by m^6^A modification, such as transcriptional regulators (Kan et al., 2017). Analysis of the functions of m^6^A-modified genes in each sample revealed that they were enriched for a large number of common functions, among which the most significant were those related to morphogenesis, development, and growth (Figure 2—figure supplement 2A). The 141 GO terms shared by the five genotypes were summarized and found to include functions about metabolism, regulation, cellular processes, signaling pathways, behavior, and immune response (Figure 2—figure supplement 2B and C). Through pathway enrichment analysis of m^6^A-modified genes, nine terms were found to be consistently enriched in all samples, including MAPK signaling pathway, Hippo signaling pathway, TGF-β pathway, etc. (Figure 2—figure supplement 2D–F).

### DMPs and their associated genes

**Figure 3.**
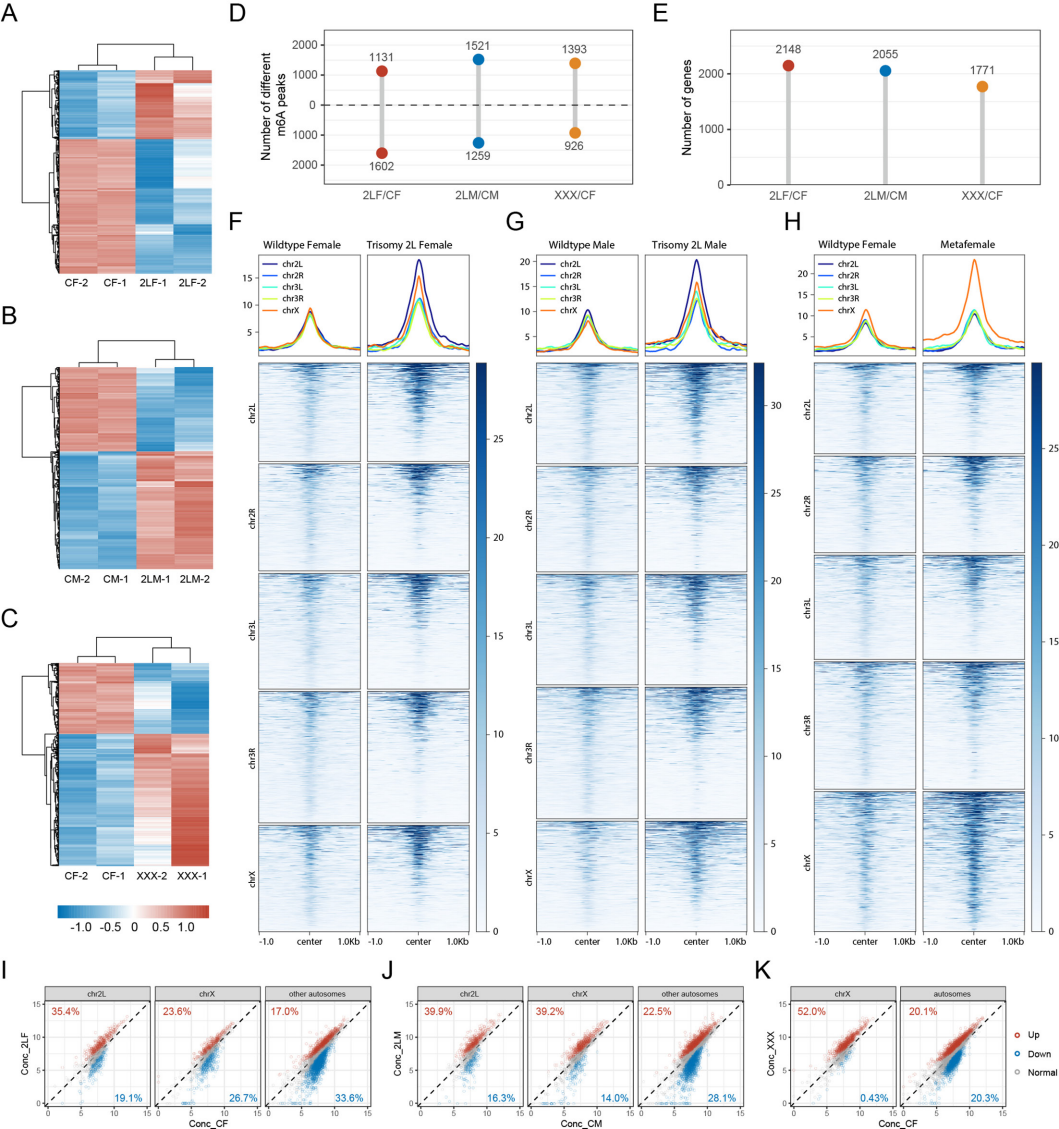
Differential m^6^A methylome analysis of aneuploid *Drosophila*. (**A–C**) Heatmaps of differentially methylated peaks (DMPs) in trisomy 2L females (**A**), trisomy 2L males (**B**), and metafemales (**C**), and their corresponding control groups. The threshold of significance was p-value≤0.1. (**D**) The number of DMPs. The threshold of significance was set to p-value≤0.1. The numbers above the horizontal dashed lines indicate peaks with up-regulated methylation levels, and the numbers below indicate peaks with down-regulated methylation levels. (**E**) The number of DMP-associated genes. (**F–H**) Profiles and heatmaps illustrating the density of m^6^A-modified reads at the DMP positions in trisomy 2L females (**F**), trisomy 2L males (**G**), metafemales (**H**), and their corresponding controls. The DMPs were divided into five groups according to the chromosomes they located. (**I–K**) Scatter plots showing the concentration of reads at methylation sites on different chromosomes in trisomy 2L females (**I**), trisomy 2L males (**J**), and metafemales (**K**). Red points indicate significantly up-regulated m^6^A peaks, blue points indicate significantly down-regulated m^6^A peaks, and gray points indicate m^6^A peaks without significant changes. The percentages of DMPs on *cis* and *trans* chromosomes were indicated in the corners of the plots. CF, wildtype female control; CM, wildtype male control; 2LF, trisomy 2L female; 2LM, trisomy 2L male; XXX, metafemale.

We then searched for the differentially methylated peaks (DMPs) in autosomal and sex chromosome aneuploidies compared with wildtype *Drosophila* of their corresponding sex. The number of DMPs and the changing trend of DMP methylation status are shown in the figures (Figure 3A–D). In trisomy 2L females, the number of up-regulated DMPs (1131) is less than that of down-regulated DMPs (1602). In trisomy 2L males, the number of up-regulated DMPs (1521) is slightly higher than that of down-regulated DMPs (1259). In metafemales, the number of up-regulated DMPs (1393) is higher than that of down-regulated DMPs (926) (Figure 3A–D). More DMP-associated genes were found in autosomal aneuploidies than in sex chromosome aneuploidy (trisomy 2L female = 2148, trisomy 2L male = 2055, metafemale = 1771; Figure 3E). Also, the total number of peaks after the differential analysis would be different from the number of m^6^A peaks identified in the previous section, because the analysis software merged and recalculated the peaks (Stark and Brown, 2013).

**Figure 3—figure supplement 1.**
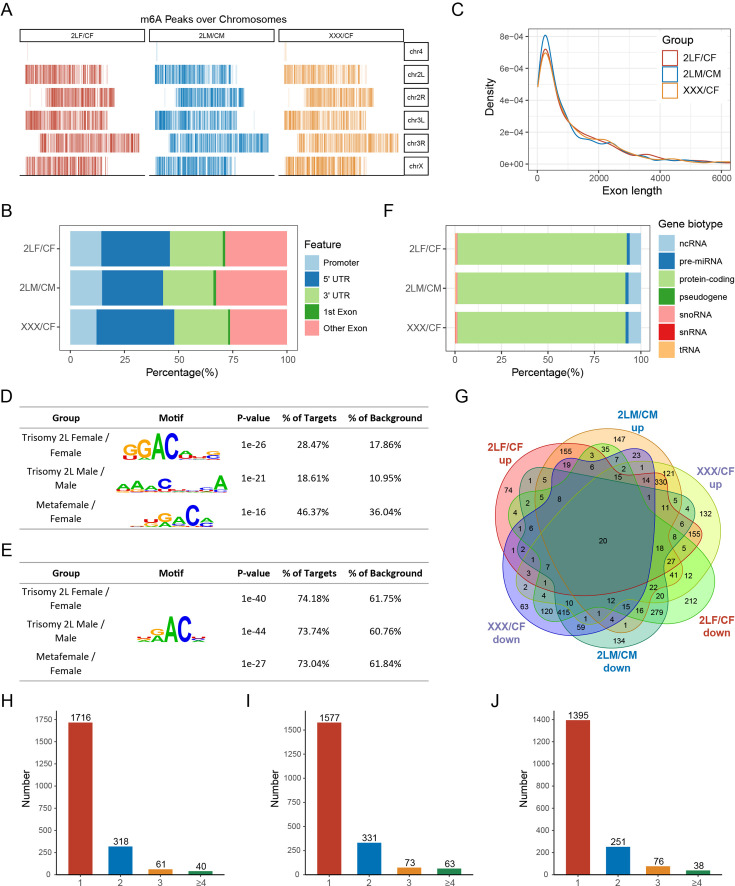
Characteristics of differentially methylated peak (DMP) associated genes. (**A**) Distribution of DMPs along each chromosome. (**B**) Percentages of DMPs localized on different gene features. (**C**) Kernel density plot showing the distribution of exon lengths of DMP-associated genes. (**D**) The top enriched motifs with de novo motif analysis of DMPs in each comparison. (**E**) Enrichment analysis performed on the known m^6^A consensus motif DRACH (D=G/A/U, R=G/A, H=U/A/C). (**F**) Gene biotypes of DMP-associated genes. (**G**) Venn diagram showing the intersection of DMP-associated genes in each comparison. (**H–J**) Statistics of the number of DMPs on DMP-associated genes in trisomy 2L females compared with wildtype females (**H**), trisomy 2L males compared with wildtype males (**I**), and metafemales compared with wildtype females (**J**). CF, wildtype female control; CM, wildtype male control; 2LF, trisomy 2L female; 2LM, trisomy 2L male; XXX, metafemale; 5′UTR, 5′ untranslated region; 3′UTR, 3′ untranslated region.

DMPs are distributed on all chromosomes (Figure 3—figure supplement 1A). Specifically, the MeRIP-Seq signal intensity at the location of DMPs showed that the m^6^A-marked reads in trisomies are denser than those of wildtypes, especially on the triple chromosomes (Figure 3F–H). The density of reads on chromosome 2L of trisomy 2L *Drosophila* is higher than that on other chromosomes, while the density of reads on chromosome X of metafemales is significantly higher than that of autosomes (Figure 3F–H). Previous studies on aneuploid *Drosophila* showed that the expression levels of genes on chromosomes with increased copy numbers were mostly similar to those in diploids, and the expression of genes on unvaried chromosomes were widely inverse regulated (Sun et al., 2013c; Birchler, 2016; Zhang et al., 2023). It can be concluded that the changes in the density of reads at DMP sites are not due to a direct gene dosage effect. Similar results were obtained by counting the methylation status of cis and trans transcripts (Figure 3I–K). Overall, the transcripts of genes located on the varied chromosomes in three trisomies have more up-regulated DMPs than down-regulated DMPs, which is more obvious in metafemales (Figure 3I–K). In trisomy 2L females and males, genes localized on other autosomes have a higher proportion of down-regulated DMPs, while the X-linked *trans* genes show sexual dimorphism and X chromosome-specific response to genomic imbalance (Figure 3I and J). For metafemales, the transcripts of *trans* genes have similar numbers of up-regulated and down-regulated DMPs (Figure 3K). Therefore, *cis* genes in trisomy generally possessed a higher proportion of up-regulated DMP-associated transcripts, whereas the methylation states of *trans* genes are variable, depending on the identities of varied chromosomes and their genomic locations. The above results give us reason to speculate that the changes of m^6^A methylation may affect dosage compensation of *cis* genes and inverse dosage modulation of *trans* genes in aneuploidy.

**Figure 4.**
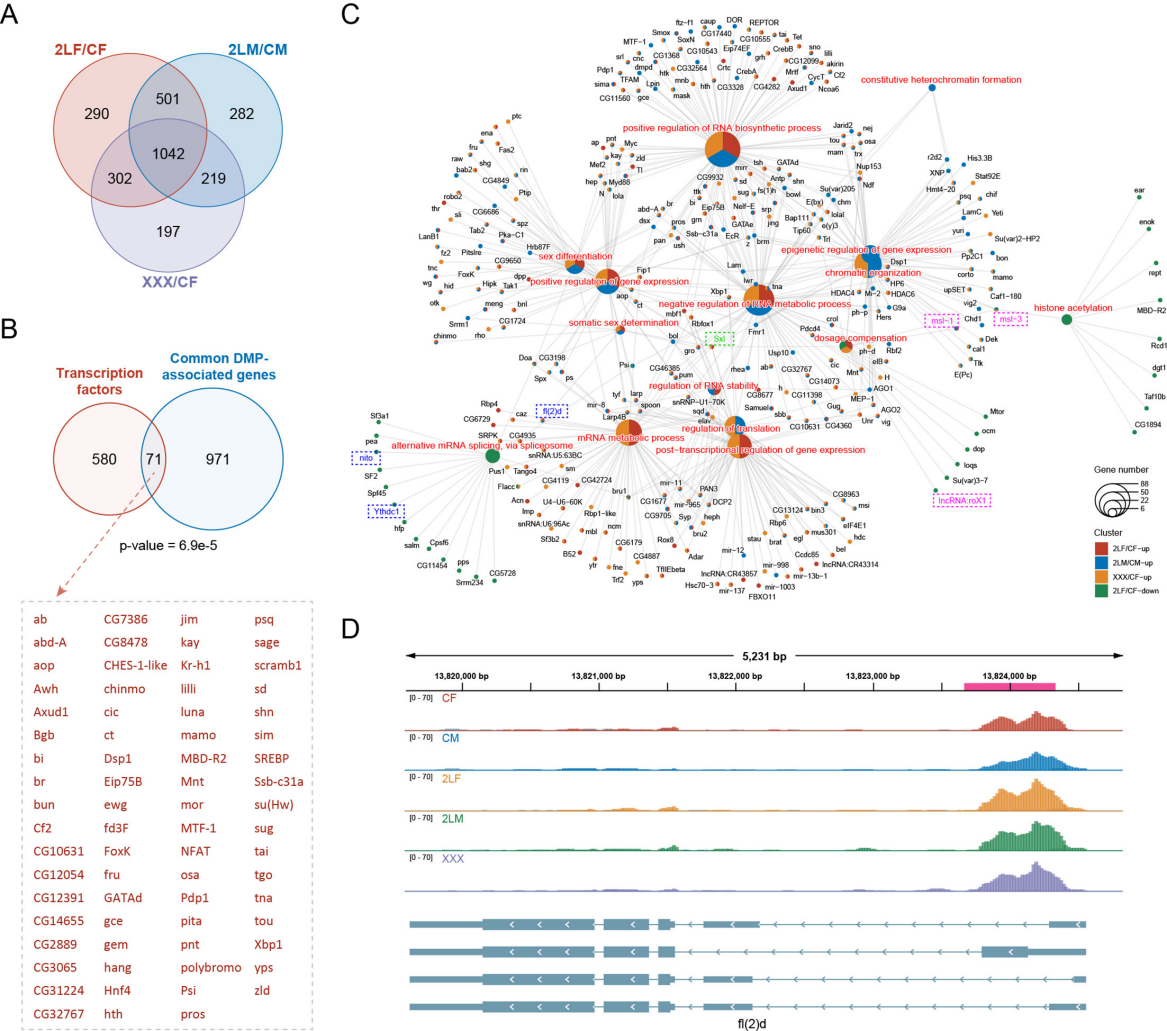
Differentially methylated peak (DMP) associated genes and their functions. (**A**) Venn diagram showing the number of common DMP-associated genes in three types of aneuploidies compared with wildtypes. (**B**) Venn diagram showing the intersection of transcription factors and the common DMP-associated genes in all comparisons. Transcription factors with DMPs were listed on the right. p-Value indicates one-tailed Fisher’s exact test. (**C**) Network showing the functions related to expression regulation and dosage compensation enriched by DMP-associated genes. The color of the nodes indicates the comparison, and the size of the function nodes represents the number of DMP-associated genes connected with them. (**D**) Genome browser example of *fl(2)d* for indicated m^6^A methylated RNA immunoprecipitation sequencing (MeRIP-seq) data. Steelblue color represents input reads, while other colors represent IP reads. Signals were displayed as the mean counts per million (CPM) of two biological replicates. The gene architectures were shown at the bottom. The magenta rectangles at above represent DMP. CF, wildtype female control; CM, wildtype male control; 2LF, trisomy 2L female; 2LM, trisomy 2L male; XXX, metafemale.

The distribution of DMPs along gene features includes 5’UTR (30–35%), 3’UTR (20–25%), promoter (10–25%), and internal exon (25–35%), among which the proportion of 3’UTR is higher than that of all m^6^A sites (Figure 3—figure supplement 1B). The length distributions of exons with DMPs are similar to that of all m^6^A-modified exons (Figure 3—figure supplement 1C). De novo motif analysis of the sequences where DMPs are located or enrichment analysis of the known motif DRACH both showed that DMP sites are enriched for the conserved motif of m^6^A (Figure 3—figure supplement 1D and E). There are also similarities between the types of DMPs-associated genes and m^6^A-marked genes (Figure 3—figure supplement 1F). The DMP-associated genes of the three aneuploidies have complex overlapping relationships (Figure 3—figure supplement 1G). Most DMP-associated genes have one differentially methylated site, and only a few genes contained more than three DMPs (Figure 3—figure supplement 1H–J). There are 1042 common DMP-associated genes in all trisomies, of which 71 were transcription factors and were significantly enriched (Fisher’s exact test p-value = 6.9e-5; Figure 4A and B). We speculate that these differentially methylated transcription factors may play an important role in the regulation of gene expression and development of aneuploidy.

**Figure 4—figure supplement 1.**
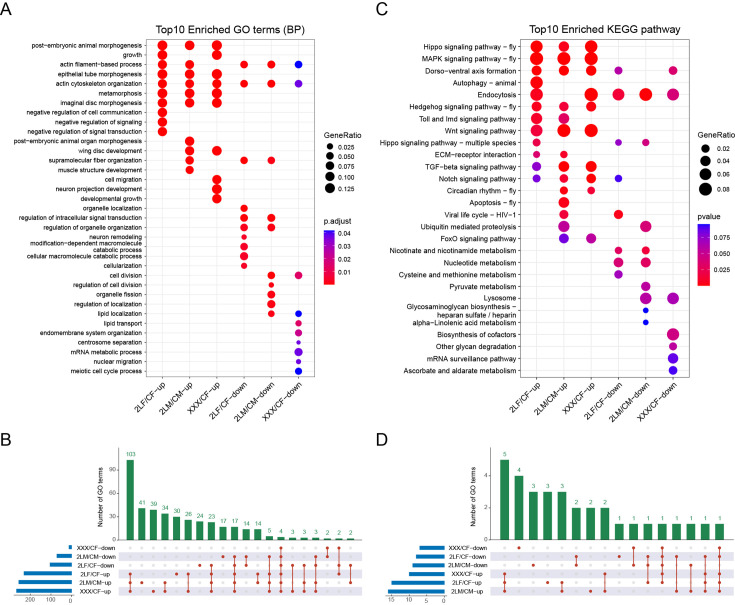
Functional and pathway analysis of differentially methylated peak (DMP) associated genes. (**A**) Functional enrichment analysis of DMP-associated genes. Top 10 enriched GO terms (Biological Process) with adjusted p-value<0.05 in each comparison were shown. (**B**) UpSet plot showing the shared sets of enriched GO terms of DMP-associated genes in different aneuploidies. (**C**) Kyoto Encyclopedia of Genes and Genomes (KEGG) pathway enrichment analysis of DMP-associated genes. Top 10 enriched pathways with p-value<0.1 in each comparison were shown. (**D**) UpSet plot showing the shared sets of enriched KEGG pathways of DMP-associated genes in different aneuploidies. CF, wildtype female control; CM, wildtype male control; 2LF, trisomy 2L female; 2LM, trisomy 2L male; XXX, metafemale.

Functional enrichment analysis showed that genes with up-regulated DMPs in three trisomies were enriched for 153 common functions, while the genes with down-regulated DMPs shared only 6 functions (Figure 4—figure supplement 1A and B). Similarly, genes with up-regulated DMPs were enriched for more consistent pathways in all aneuploids (Figure 4—figure supplement 1C and D). Most of the functions of DMP-associated genes are the same as those of m^6^A-marked genes, and these genes are also enriched for cell fate commitment, dorsal/ventral pattern formation, sex differentiation, post-transcriptional regulation of gene expression, and other additional GO terms. We found that many DMP-associated genes have functions related to gene expression regulation and dosage compensation, including some components of MSL complex (*msl-1*, *msl-3*, *lncRNA:roX1*) and the major sex-determining factor *Sxl* (Figure 4C). We also noticed that m^6^A component genes *fl(2)d*, *nito*, and *Ythdc1* themselves are differentially methylated in aneuploidy (Figure 4C), e.g., there was a common significantly up-regulated m^6^A peak in the 5’UTR of the transcripts of *fl(2)d* in three aneuploidies (Figure 4D). These data suggest that m^6^A dynamics in aneuploids may be involved in the expression regulation of unbalanced genomes through various genes with regulatory functions.

### Relationships between m^6^A and dosage-related modulation of gene expression in aneuploidy

**Figure 5.**
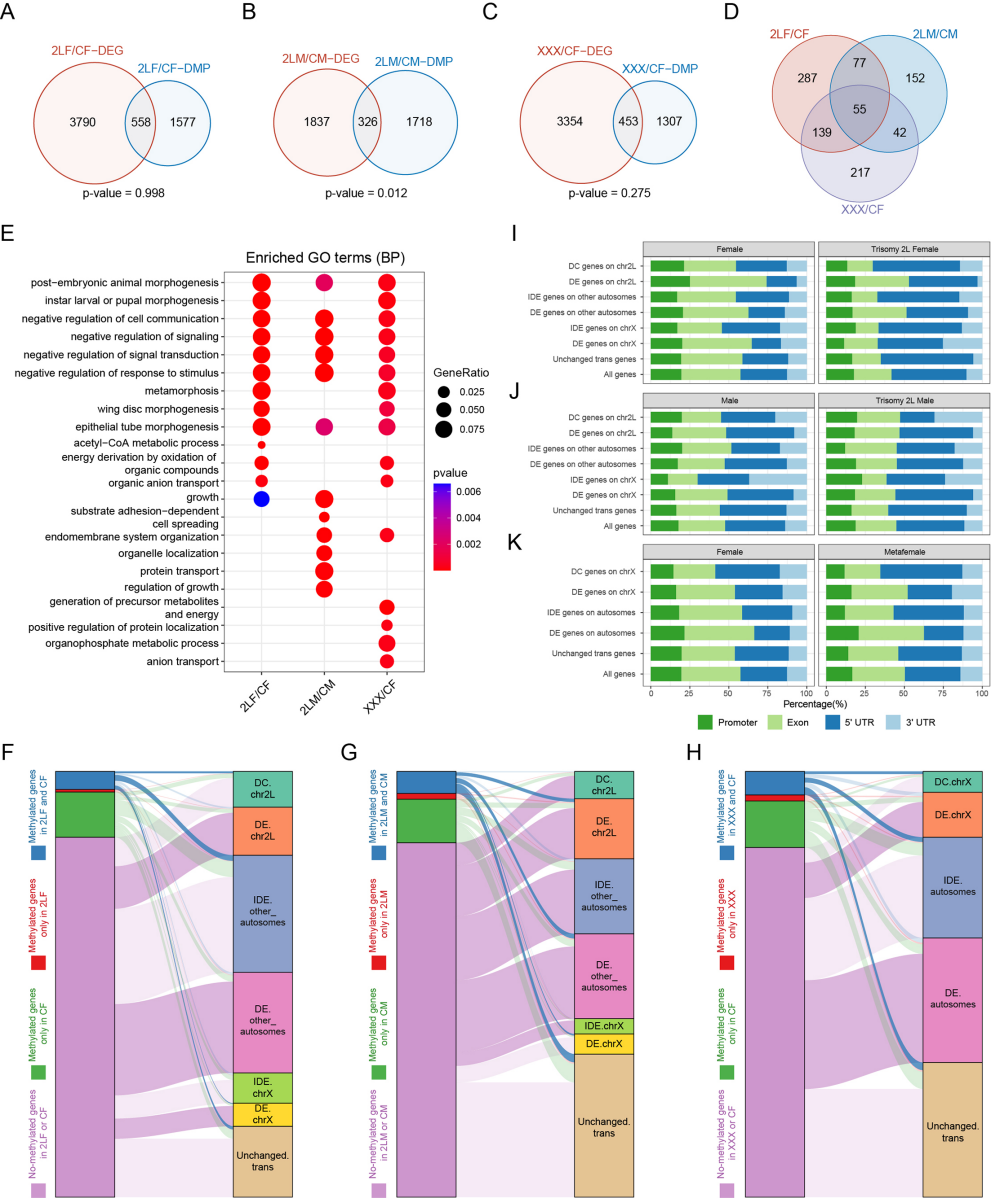
Relationships between RNA m^6^A methylation and gene expression in aneuploidy. (**A–C**) Venn diagrams showing the intersections of differentially expressed genes (DEGs) and differentially methylated peak (DMP) associated genes in trisomy 2L females (**A**), trisomy 2L males (**B**), and metafemales (**C**) compared with their corresponding controls. p-Values indicate one-tailed Fisher’s exact tests. (**D**) The common differentially expressed and differentially methylated genes in all groups. (**E**) Functional enrichment analysis of simultaneously differentially expressed and differentially methylated genes. Top 10 enriched GO terms (Biological Process) with p-value<0.1 in each comparison were shown. (**F–H**) Sankey diagrams showing the relationships between genes with different m^6^A-modified states and genes with canonical dosage effect (DE), dosage compensation (DC), and inverse dosage effect (IDE) in trisomy 2L females (**F**), trisomy 2L males (**G**), and metafemales (**H**). Enrichment analysis was performed on each two groups of genes, and deep color lines indicate significant connection relationships (Fisher’s exact test p-value<0.05). (**I–K**) Gene feature distributions for m^6^A peaks on genes with canonical DE, DC, and IDE in trisomy 2L females (**I**), trisomy 2L males (**J**), metafemales (**K**), and their corresponding controls. CF, wildtype female control; CM, wildtype male control; 2LF, trisomy 2L female; 2LM, trisomy 2L male; XXX, metafemale; DEG, differentially expressed gene; DMP, differentially methylated peak; DE, dosage effect; DC, dosage compensation; IDE, inverse dosage effect. Canonical DE refers to ratio >1.25, DC stands for 0.8<ratio<1.25, IDE stands for 0.5<ratio<0.8, and unchanged refers to 0.8<ratio<1.25.

To explore the relationships between RNA m^6^A modification and gene expression regulation under genomic imbalance, we combined the results of MeRIP-Seq and RNA-seq, and performed a comprehensive analysis. The results showed that about 12–15% of genes differentially expressed in aneuploid *Drosophila* also belong to DMPs-associated genes (Figure 5A–C). However, except for trisomy 2L males, the DEGs of the other two trisomies did not show enrichment of differential m^6^A methylation (Fisher’s exact test p-values: trisomy 2L male = 0.012, trisomy 2L female and metafemale >0.05). There are 55 genes that are both differentially expressed and differentially methylated in all aneuploidies (Figure 5D). By analyzing the functions of genes that are simultaneously differentially expressed and differentially methylated, we obtained a number of consistent biological functions, including post-embryonic animal morphogenesis, cell communication, signal transduction, response to stimulation, and so on (Figure 5E), possibly reflecting the important roles of m^6^A-regulated expression in cell signaling and development of organisms.

**Figure 5—figure supplement 1.**
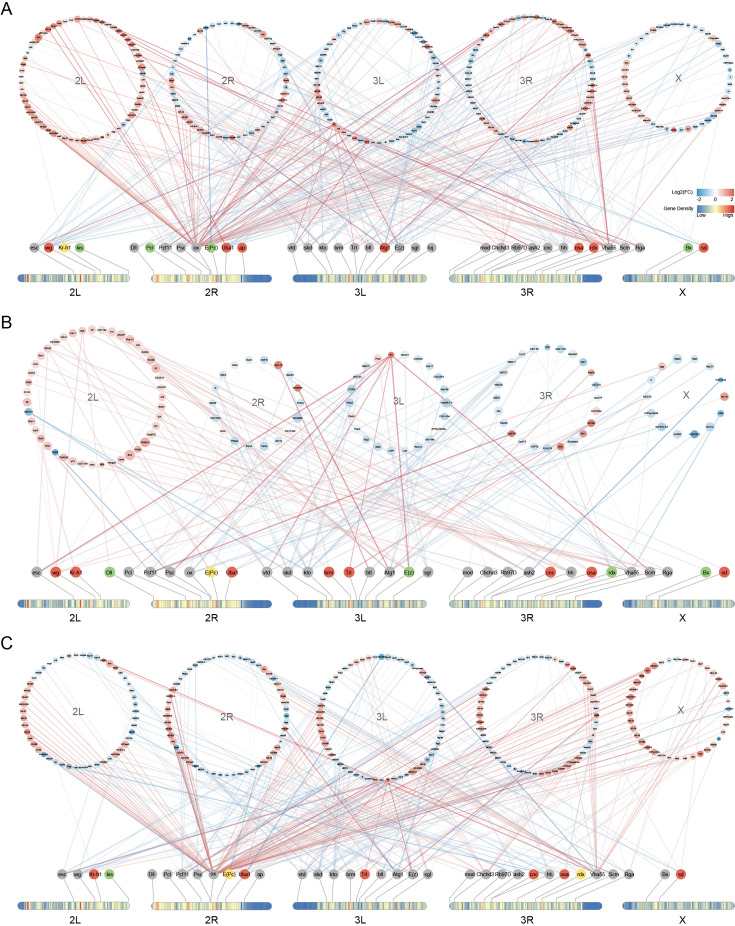
Dosage-sensitive modifiers and their interactors in aneuploid *Drosophila*. (**A–C**) Networks showing the methylation status of known dosage-sensitive modifiers and the expression changes of genes interact with these modifiers in trisomy 2L females (**A**), trisomy 2L males (**B**), and metafemales (**C**), respectively. Protein-protein interaction (PPI) relationships were obtained from the STRING database. The colors of the heatmaps on chromosomes represent the gene density. Dosage-sensitive modifiers were arranged according to their localizations, with red indicating genes with up-regulated differentially methylated peaks (DMPs), green indicating genes with down-regulated DMPs, yellow indicating genes with both up- and down-regulated DMPs, and gray indicating genes without DMPs. The interactors of these modifiers were also separated by chromosomes, and the colors of the nodes and edges indicate the log2(fold changes) of gene expression in trisomies. The width of the edges represents the combined score, and only interactions with combined score ≥700 were selected for presentation.

By analyzing the crossover of different groups of m^6^A-modified genes and genes with canonical dosage effect, dosage compensation, and inverse dosage effect, we further revealed the relationships between m^6^A-modified genes and dosage-related effects in aneuploidy (Figure 5F–H). The results showed that in trisomy 2L females, *cis* dosage compensation genes, *trans* autosomal dosage effect genes, and *trans* unchanged genes are significantly enriched in m^6^A group with methylation in both trisomy and control; meanwhile, *cis* dosage effect genes, other autosomal dosage effect genes, and X-linked dosage effect genes are enriched in the group without methylation in both trisomy and control (Figure 5F). On the contrary, in trisomy 2L males, *cis* dosage effect genes, *trans* dosage effect genes, and *trans* unchanged genes are significantly enriched in m^6^A group whose genes are methylated in both trisomy and control; while the group of genes that are not methylated at all is enriched for *cis* dosage effect genes, *cis* dosage compensation genes, *trans* inverse dosage effect genes, and other autosomal dosage effect genes (Figure 5G). Metafemales performed similarly to trisomy 2L female, with significant enrichment of *cis* dosage compensation genes, autosomal *trans* dosage effect genes, and *trans* unchanged genes in m^6^A group where both trisomy and control genes are methylated; and the group without methylation is enriched for *cis* dosage effect genes and *trans* dosage effect genes (Figure 5H). These results indicated that m^6^A-modified genes in aneuploid *Drosophila* females are mainly related to dosage compensation and inverse dosage effect, while genes not modified by m^6^A are mainly related to direct dosage effect. Male aneuploids did not follow this trend.

Furthermore, the distributions of m^6^A modification sites along gene features in genes with classical dosage-related effects were also studied (Figure 5I–K). We found that in wildtype females, trisomy 2L females, and metafemales, a higher proportion of m^6^A sites on genes with canonical dosage compensation and inverse dosage effect are distributed in the 5’UTR than genes with dosage effects (Figure 5I and K). At the same time, this proportion is higher in trisomy than in wildtype. In contrast to females, trisomy 2L males have a higher proportion of 5’UTR RNA m^6^A modification on dosage effect genes (Figure 5J). The above study again demonstrates the sexual dimorphism of RNA m^6^A modification in response to aneuploidy. In addition, it has been suggested that m^6^A residues in the 5’UTR region may have unique regulatory functions (Luo et al., 2014; Meyer and Jaffrey, 2014). Therefore, we hypothesized that the high level of 5’UTR m^6^A modification on genes with dosage compensation and inverse dosage effect might regulate the down-regulation of these genes.

Dozens of dosage-sensitive modifiers have been identified in *Drosophila*, whose dosage changes can negatively or positively regulate the gene expression across the whole genome like aneuploidy (Birchler et al., 2001; Birchler and Veitia, 2007). Protein-protein interaction (PPI) networks between dosage-sensitive modifiers and differentially expressed genes (DEGs) were constructed to investigate whether RNA m^6^A modification could participate in the gene regulatory networks in unbalanced genomes through these regulators (Figure 5—figure supplement 1A–C). In the three types of aneuploidies, there are more dosage-sensitive modifiers with up-regulated DMPs than with down-regulated DMPs. The regulators *ox* and *Vha55* without DMPs interact with a large number of significantly up-regulated DEGs in trisomy 2L females and metafemales, whereas these two regulators interact with a small number of down-regulated DEGs in trisomy 2L males. Up-regulated DMP-associated regulators *wg*, *Uba1*, *ap*, *Atg1*, *osa*, *rdx*, and *sd* in trisomy 2L females mainly interacted with significantly down-regulated DEGs (Figure 5—figure supplement 1A); and up-regulated DMP-associated regulators *Uba1*, *osa*, and *sd* in metafemales also interacted with down-regulated DEGs (Figure 5—figure supplement 1C). Up-regulated DMP-associated regulators *wg*, *Kr-h1*, and *Trl* mainly interacted with up-regulated DEGs in trisomy 2L males (Figure 5—figure supplement 1B). In addition, a preponderance of significantly down-regulated DEGs interacted with dosage-sensitive modifiers is observed on *trans* chromosomes of all *Drosophila* trisomies (Figure 5—figure supplement 1A–C).

### Alterations of m^6^A may be involved in differential alternative splicing in imbalanced genomes

**Figure 6.**
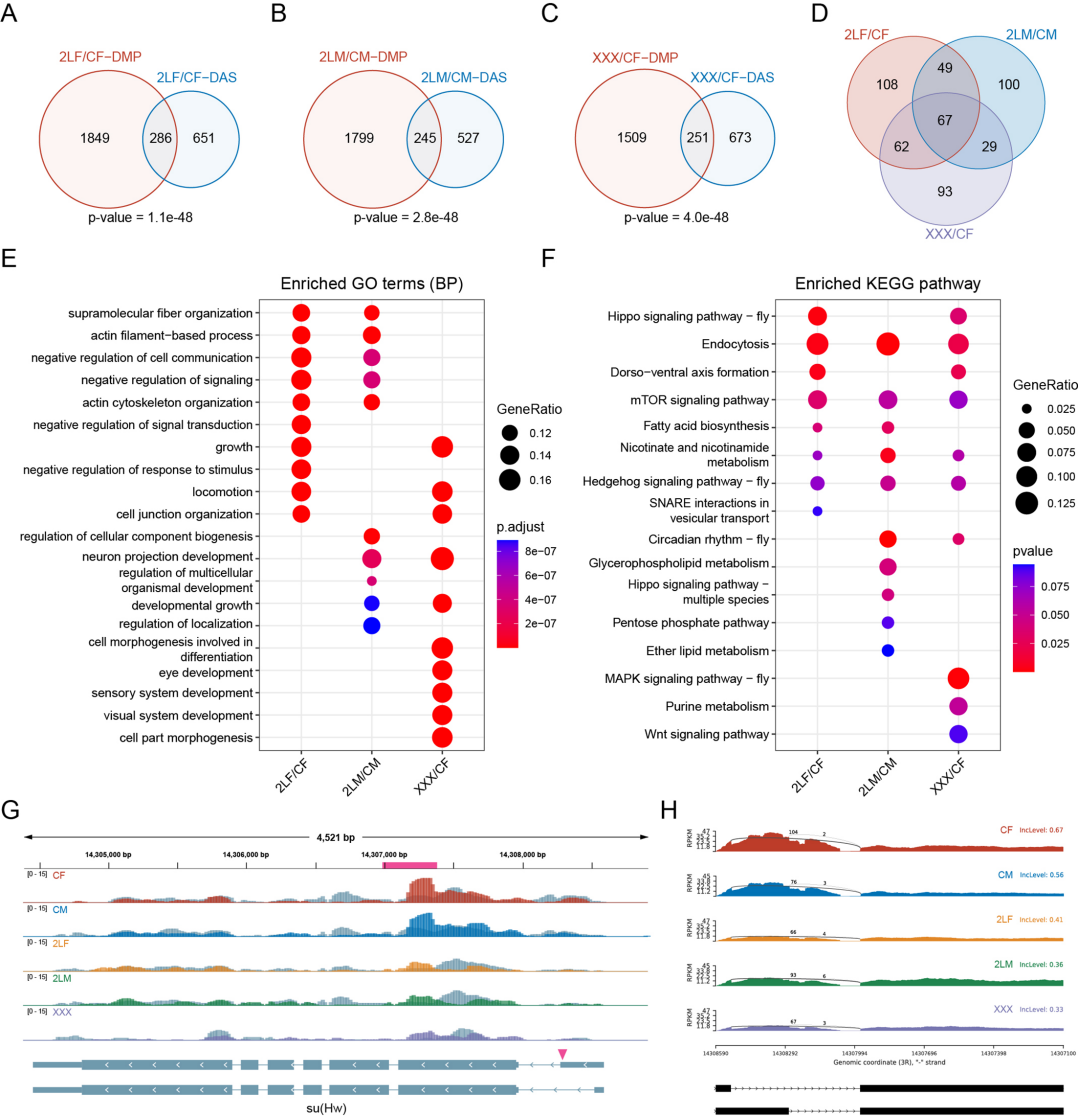
Combined analysis of differential alternative splicing (DAS) and differential methylation. (**A–C**) Venn diagrams showing the intersections of DAS genes and differentially methylated peak (DMP) associated genes in trisomy 2L females (**A**), trisomy 2L males (**B**), and metafemales (**C**) compared with their corresponding controls. p-Values indicate one-tailed Fisher’s exact tests. (**D**) The common differentially alternatively spliced and differentially methylated genes in all groups. (**E**) Functional enrichment analysis of simultaneously differentially alternatively spliced and differentially methylated genes. Top 10 enriched GO terms (Biological Process) with p-value<0.05 in each comparison were shown. (**F**) Kyoto Encyclopedia of Genes and Genomes (KEGG) pathway enrichment analysis of simultaneously differentially alternatively spliced and differentially methylated genes. Top 10 enriched pathways with p-value<0.1 in each comparison were shown. (**G**) Genome browser example of *su(Hw*) for indicated m^6^A methylated RNA immunoprecipitation sequencing (MeRIP-Seq) data. Steelblue color represents input reads, while other colors represent IP reads. Signals were displayed as the mean CPM of two biological replicates. The gene architecture was shown at the bottom (only two representative transcript isoforms were shown). The magenta rectangle at above represents DMP. The magenta arrowhead indicates the position of differential alternative splicing. (**H**) Sashimi plot depicting RNA sequencing reads and exon junction reads at the position where the differential splicing events occur on *su(Hw*). The gene model was shown below. One of the biological replicates was chosen for representation. CF, wildtype female control; CM, wildtype male control; 2LF, trisomy 2L female; 2LM, trisomy 2L male; XXX, metafemale; DAS, differential alternative splicing; DMP, differentially methylated peak; CPM, counts per million; RPKM, reads per kilobase per million mapped reads.

Next, we analyzed the differential alternative splicing events in aneuploid *Drosophila*. More than 1000 differential splicing events have been identified in different aneuploids, and about one-third of them are of the type of skipped exon (SE) (Figure 6—figure supplement 1A–C). The biological functions of differentially spliced transcripts are mainly involved in macromolecular fiber organization, locomotion, and growth (Figure 6—figure supplement 1D), and these genes are enriched in heterogeneous pathways in different aneuploidies (Figure 6—figure supplement 1E). Notably, we found that genes with DMPs are significantly enriched for differential alternative splicing events in three kinds of aneuploid *Drosophila* (Fisher’s exact test p-values<0.05; Figure 6A–C). Among the genes whose transcripts are differentially spliced, 27–32% are also differentially methylated (Figure 6A–C). There are 67 genes with both differential alternative splicing and differential m^6^A methylation in all aneuploidies (Figure 6D). The functions of these genes are similar to those of all differentially spliced genes (Figure 6E), but more consistent Kyoto Encyclopedia of Genes and Genomes (KEGG) pathways are enriched, including endocytosis, mTOR signaling pathway, Hedgehog signaling pathway, and so on (Figure 6F).

**Figure 6—figure supplement 1.**
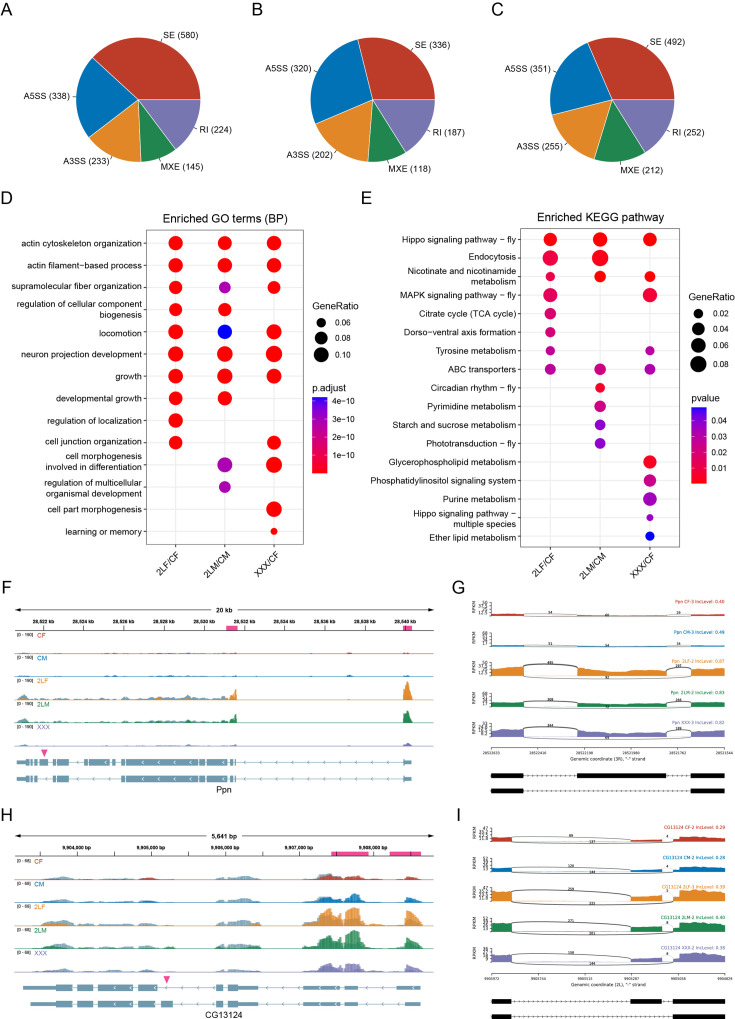
Alternative splicing analysis in aneuploidies. (**A–C**) Numbers of differential alternative splicing events in trisomy 2L females (**A**), trisomy 2L males (**B**), and metafemales (**C**). SE, skipped exon; A5SS, alternative 5’ splice site; A3SS, alternative 3’ splice site; MXE, mutually exclusive exons; RI, retained intron. (**D**) Functional enrichment analysis of differentially alternatively spliced genes. Top 10 enriched GO terms (Biological Process) with p-value<0.05 in each comparison were shown. (**E**) Kyoto Encyclopedia of Genes and Genomes (KEGG) pathway enrichment analysis of differentially alternatively spliced genes. Top 10 enriched pathways with p-value<0.05 in each comparison were shown. (**F,H**) Genome browser example of *Ppn* (**F**) and *CG13124* (**H**) for indicated m^6^A methylated RNA immunoprecipitation sequencing (MeRIP-seq) data. Steelblue color represents input reads, while other colors represent IP reads. Signals were displayed as the mean CPM of two biological replicates. The gene architectures were shown at the bottom (only two representative transcript isoforms were shown). The magenta rectangles at above represent differentially methylated peaks (DMPs). The magenta arrowheads indicate the positions of differential alternative splicing. (**G,I**) Sashimi plots depicting RNA sequencing reads and exon junction reads at the positions where the differential splicing events occur on *Ppn* (**G**) and *CG13124* (**I**). The gene models were shown below. One of the biological replicates was chosen for representation. CF, wildtype female control; CM, wildtype male control; 2LF, trisomy 2L female; 2LM, trisomy 2L male; XXX, metafemale; CPM, counts per million; RPKM, reads per kilobase per million mapped reads.

In all trisomy *Drosophila*, 10 genes are shown to be differentially expressed, with their transcripts also being differentially spliced and differentially methylated. Three of them are illustrated below [*su(Hw*), *Ppn*, and *CG13124*]. *su(Hw*) is a component of the gypsy chromatin insulator complex, which is a regulatory element that establishes independent domains of transcriptional activity (Roseman et al., 1993). Due to the close relationship between *su(Hw*) and second-site modifiers (Rabinow and Birchler, 1989), and *BEAF-32*, which is also an insulator DNA-binding protein, has been proposed as a possible inverse dosage regulator (Gurudatta et al., 2012; Zhang et al., 2021b). We speculated that the transcription factor *su(Hw*) may also be a dosage-sensitive regulator. The data showed that the transcription levels of *su(Hw*) are up-regulated in all three aneuploidies, and there is a consistent m^6^A modification site with significantly down-regulated methylation (Figure 6G). At the same time, the transcripts of this gene have a common alternative 5’ splice site (A5SS), and its inclusion levels in trisomies are down-regulated, i.e., more short transcript isoforms are generated (Figure 6G). *Ppn* gene encodes an essential extracellular matrix protein that influences cell rearrangements. The expression level of *Ppn* and its m^6^A methylation in 5’UTR region are both significantly up-regulated in trisomies (Figure 6—figure supplement 1F). In addition, an alternatively spliced exon is significantly less frequently skipped in all aneuploid *Drosophila* (Figure 6—figure supplement 1G). The third gene, *CG13124*, which may be involved in regulation of translational initiation, is up-regulated in aneuploids. Two m^6^A sites in its 5’UTR region are methylated at higher levels in all aneuploid *Drosophila* (Figure 6—figure supplement 1H). Its transcripts also have a common significantly different exon-skipping event (Figure 6—figure supplement 1I).

These results indicate that there are complicated relationships among RNA m^6^A modification, gene expression, and alternative splicing under the condition of genome imbalance. RNA splicing seems to be more closely related to m^6^A methylation than gene transcription. The m^6^A sites located in 5’UTR show remarkable changes in methylation levels in aneuploid *Drosophila*, and may be involved in the regulation of some differential alternative splicing events, such as exon skipping.

### Interactions between m^6^A and *Drosophila* MSL complex

**Figure 7.**
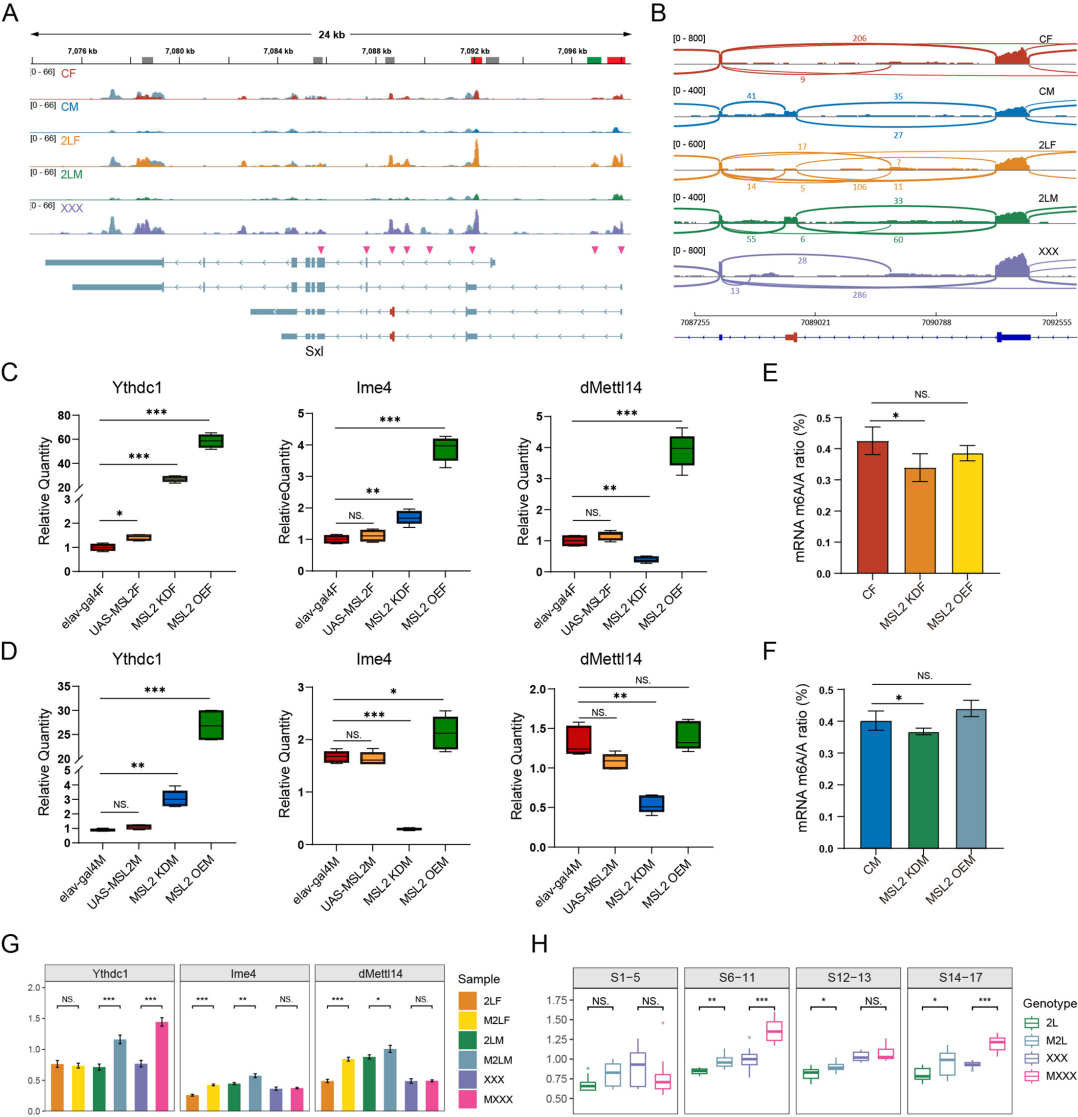
Interactions between m^6^A and *Drosophila* male-specific lethal (MSL) complex. (**A**) Genome browser example of *Sxl* for indicated m^6^A methylated RNA immunoprecipitation sequencing (MeRIP-Seq) data. Steelblue color represents input reads, while other colors represent IP reads. Signals were displayed as the mean counts per million (CPM) of two biological replicates. The gene architecture was shown at the bottom (only four representative transcript isoforms were shown). The rectangles at above represent m^6^A peaks, where red indicates up-regulated differentially methylated peaks (DMPs), green indicates down-regulated DMP, and gray indicates no significant changes. The magenta arrowheads indicate the positions of alternative splicing. (**B**) Sashimi plot depicting RNA sequencing reads and exon junction reads at the position where the differential splicing events occurs on *Sxl*. The gene model was shown below, with the third exon indicated in red. One of the biological replicates was chosen for representation. CF, wildtype female control; CM, wildtype male control; 2LF, trisomy 2L female; 2LM, trisomy 2L male; XXX, metafemale. (**C,D**) RT-qPCR analysis of messenger RNA (mRNA) levels of m^6^A components in the heads of MSL2 transgenic female (**C**) and male (**D**) *Drosophila* adults. (**E,F**) Abundance of mRNA m^6^A modification in the heads of MSL2 transgenic females (**E**) and males (**F**). MSL2 KDF, MSL2 neural-knockdown female; MSL2 KDM, MSL2 neural-knockdown male; MSL2 OEF, MSL2-overexpressed female; MSL2 OEM, MSL2-overexpressed male. Sample size = 3. Student’s *t* test *p<0.05, **p<0.01, ***p<0.001. (**G**) RT-qPCR analysis of mRNA levels of m^6^A regulators in the brains of trisomy and MSL2-overexpressed trisomy *Drosophila* larvae. 2LF, trisomy 2L female; 2LM, trisomy 2L male; XXX, metafemale; M2LF, MSL2-overexpressed trisomy 2L female; M2LM, MSL2-overexpressed trisomy 2L male; MXXX, MSL2-overexpressed metafemale. Sample size = 3. Student’s *t* test *p<0.05, **p<0.01, ***p<0.001. (**H**) The expression levels of Ime4 in trisomy and MSL2-overexpressed trisomy embryos represented by relative fluorescence intensity of probes. The expression of wildtype embryos was set as one. 2L, trisomy 2L; M2L, MSL2-overexpressed trisomy 2L; XXX, metafemale; MXXX, MSL2-overexpressed metafemale. Sample size = 10. Student’s *t* test *p<0.05, **p<0.01, ***p<0.001.

Previous studies have shown that m^6^A components are involved in regulating the alternative splicing of sex-determining gene *Sxl* in *Drosophila*, and the deficiency of m^6^A writers or readers will lead to the reduction of female-specific isoforms of *Sxl* (Haussmann et al., 2016; Lence et al., 2016; Kan et al., 2017). *Sxl* is also a direct target of RNA m^6^A modification (Kan et al., 2017). We found that *Sxl* transcripts are both differentially methylated and differentially spliced in three kinds of aneuploid *Drosophila* (Figure 7A and B). For trisomy 2L females and metafemales, two common m^6^A peaks are significantly up-regulated in the 5’UTR region of *Sxl*. However, trisomy 2L males have a significantly down-regulated m^6^A peak in the 5’UTR (Figure 7A). Meanwhile, multiple junctions of *Sxl* transcripts undergo complicated alternative splicing in aneuploid *Drosophila*, including SE, A5SS, alternative 3’ splice site (A3SS), and mutually exclusive exons types (Figure 7A). By checking the distributions of RNA sequencing (RNA-seq) reads near the male-specific exon (namely the third exon), it can be observed that there are almost no mapped reads on the third exon in wildtype females, trisomy 2L females, and metafemales, while the reads mapped to the second and fourth exons are highly prevalent (Figure 7B). On the contrary, wildtype males and trisomy 2L males have a substantial number of reads on the third exon, accompanied by a smaller number of reads on the adjacent two exons (Figure 7B). Notably, we found that a small number of RNA-seq reads aligned to the male-specific exon appeared in trisomy 2L females, which was identified as an SE-type differential alternative splicing event (FDR = 7.4e-7; Figure 7B). This variation may be related to the abnormal m^6^A methylation levels under the condition of genomic imbalance.

**Figure 7—figure supplement 1.**
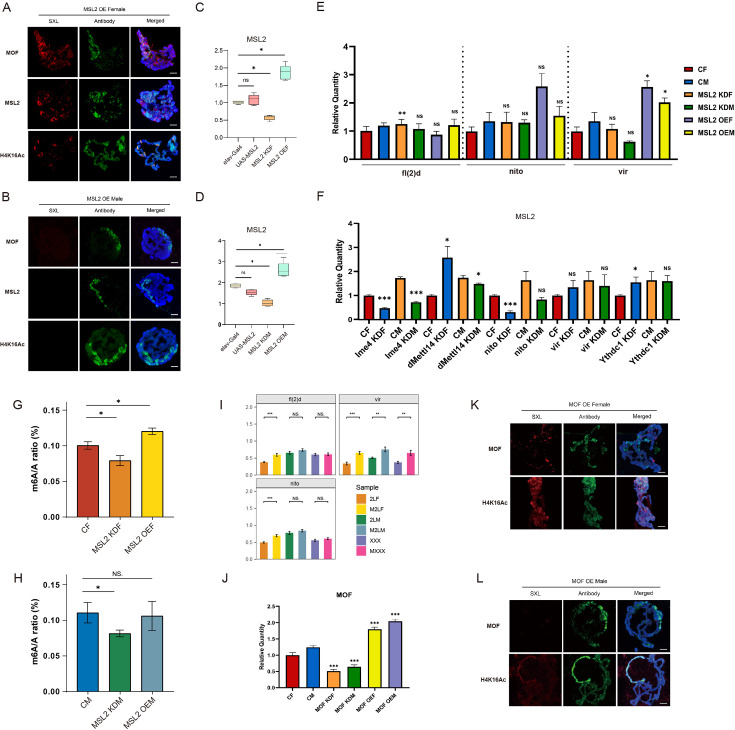
The relationships between m^6^A methylation and male-specific lethal (MSL) complex. (**A,B**) Immunofluorescence of polytene chromosomes in MSL2-overexpressed female (**A**) and male (B) larvae. The red channel is the signal from SXL and the green channel is the signal from antibodies of MSL complex components. DNA is stained with DAPI in blue. Scale bars, 10 μm. OE, overexpressed. (**C,D**) RT-qPCR analysis of messenger RNA (mRNA) levels of MSL2 in the heads of MSL2 transgenic female (C) and male (**D**) *Drosophila* adults. Sample size = 3. Student’s *t* test *p<0.05, **p<0.01, ***p<0.001. OE, overexpressed. (**E**) RT-qPCR analysis of mRNA levels of m^6^A regulators in the heads of MSL2 transgenic *Drosophila*. CF, wildtype female control; CM, wildtype male control; OEF, overexpressed female; OEM, overexpressed male; KDF, knockdown female; KDM, knockdown male. Sample size = 3. Student’s *t* test *p<0.05, **p<0.01, ***p<0.001. (**F**) RT-qPCR analysis of mRNA levels of MSL2 in the heads of m^6^A regulators knockdown *Drosophila*. CF, wildtype female control; CM, wildtype male control; OEF, overexpressed female; OEM, overexpressed male; KDF, knockdown female; KDM, knockdown male. Sample size = 3. Student’s *t* test *p<0.05, **p<0.01, ***p<0.001.(**G,H**) Abundance of total RNA m^6^A modification in the heads of MSL2 transgenic females (**G**) and males (**H**). MSL2 KDF, MSL2 neural-knockdown female; MSL2 KDM, MSL2 neural-knockdown male; MSL2 OEF, MSL2-overexpressed female; MSL2 OEM, MSL2-overexpressed male. Sample size = 3. Student’s *t* test *p<0.05, **p<0.01, ***p<0.001.(**I**) RT-qPCR analysis of mRNA levels of m^6^A regulators in the brains of trisomy and MSL2-overexpressed trisomy *Drosophila* larvae. 2LF, trisomy 2L female; 2LM, trisomy 2L male; XXX, metafemale; M2LF, MSL2-overexpressed trisomy 2L female; M2LM, MSL2-overexpressed trisomy 2L male; MXXX, MSL2-overexpressed metafemale. Sample size = 3. Student’s *t* test *p<0.05, **p<0.01, ***p<0.001. (**J**) RT-qPCR analysis of mRNA levels of MOF in the heads of MOF transgenic *Drosophila*. CF, wildtype female control; CM, wildtype male control; OEF, overexpressed female; OEM, overexpressed male; KDF, knockdown female; KDM, knockdown male. Sample size = 3. Student’s *t* test *p<0.05, **p<0.01, ***p<0.001. (**K,L**) Immunofluorescence of polytene chromosomes in MOF-overexpressed female (**H**) and male (**I**) larvae. The red channel is the signal from SXL and the green channel is the signal from MOF or H4K16Ac. DNA is stained with DAPI in blue. Scale bars, 10 μm. OE, overexpressed.

The abnormal expression of m^6^A components reduces the survival of female *Drosophila*, which is thought to be probably caused by the expression of downstream MSL complex (Haussmann et al., 2016). To investigate the interplay between the MSL complex and m^6^A modification, we examined the responses of m^6^A regulators in transgenic *Drosophila* strains with MSL2 mutation or overexpression (Figure 7—figure supplement 1A–E; Figure 7C and D). The results revealed significant changes in the expression profiles of m^6^A regulators in MSL2 transgenic strains, especially the m^6^A reader protein Ythdc1, which increased tens of fold in MSL2 knockdown and overexpressed *Drosophila* (Figure 7C and D; Figure 7—figure supplement 1E). We also observed that the trends of Ime4 expression in females and males were the same in MSL2-overexpressed *Drosophila*, whereas there was obvious sexual dimorphism in MSL2-knockdown samples (Figure 7C and D), which may be due to the ectopic assembly of MSL complex in MSL2-overexpressed females (Zhang et al., 2021a). In addition, we also examined the expression levels of MSL2 when *Ythdc1* was knocked down, and found that MSL2 was also significantly increased in females (Figure 7—figure supplement 1F). All these results strongly suggest a potential relationship between MSL2 and Ythdc1. Next, we further compared the overall abundance of m^6^A on mRNA in MSL2 transgenic and wildtype *Drosophila* (Figure 7E and F). The results showed that mRNA m^6^A levels were significantly decreased with MSL2 knockdown in females and males, but overexpression of MSL2 failed to exert a discernible effect on m^6^A abundance (Figure 7E and F).

In the next, we investigated the expression of m^6^A regulators in aneuploid *Drosophila* overexpressing MSL2, according the results that the MSL complex could be regulated directly or indirectly by m^6^A modification, and the sexual dimorphism of RNA m^6^A modification in response to aneuploidy. It is found that the transcription levels of m^6^A regulators are significantly up-regulated in the brains of most aneuploid larvae that overexpressed MSL2 (Figure 7G; Figure 7—figure supplement 1I). These results are not completely consistent with the quantitative results when MSL2 was overexpressed in diploids, which may be related to the effect of unbalanced genomes. We also used TSA-FISH to detect the expression and distribution of m^6^A components during embryogenesis in aneuploidies overexpressing MSL2 (Figure 7H; Figure 1—figure supplement 2). The subembryonic and subcellular distributions of mRNAs for m^6^A methyltransferases and reading protein did not appear to be affected by ectopic expression of MSL2 in aneuploid *Drosophila* embryos (Figure 1—figure supplement 2A–F; Figure 1—figure supplement 2A’–F’). But the relative expression of m^6^A components showed diverse dynamics, among which the levels of the most important methyltransferase Ime4 are significantly up-regulated in autosomal trisomy and sex chromosome trisomy with MSL2 overexpression (Figure 7H; Figure 1—figure supplement 2A”–F”). Considering that unbalanced genomes can affect the expression of MSL complex subunits, and the above results show that there is a close relationship between MSL complex and m^6^A modification, we speculate that unbalanced genomes may influence the expression of m^6^A regulators, possibly through the MSL complex.

### Relationship of H4K16Ac with m^6^A modification

**Figure 8.**
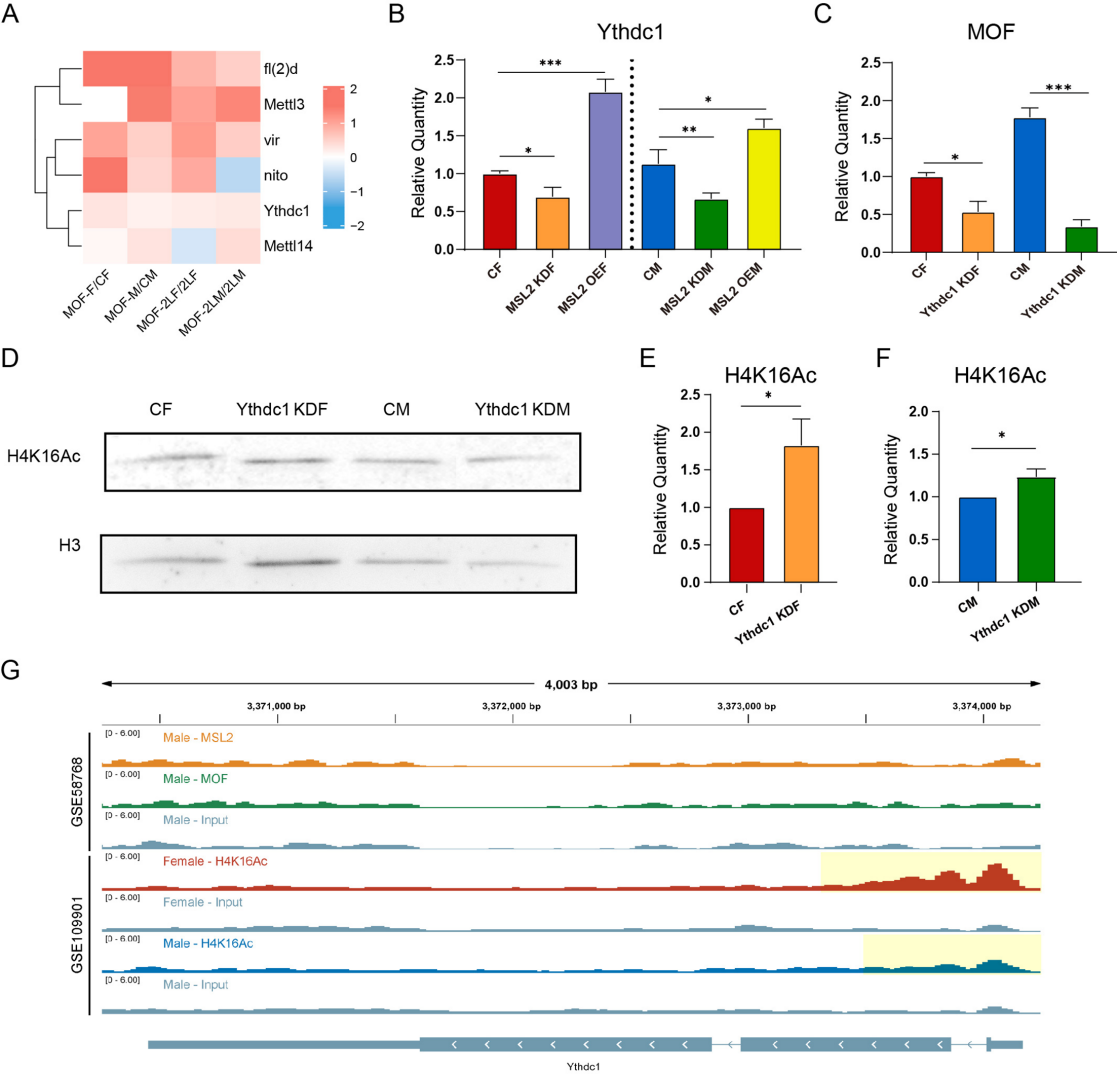
RNA m^6^A modification regulate histone acetyltransferase MOF and H4K16Ac. (**A**) Heatmap of the expression changes of m^6^A regulators in MOF overexpressing *Drosophila* larvae. The color of the heatmap represents log2(ratio). CF, wildtype female control; CM, wildtype male control; MOF-F, MOF-overexpressed female; MOF-M, MOF-overexpressed male; 2LF, trisomy 2L female; 2LM, trisomy 2L male; MOF-2LF, MOF-overexpressed trisomy 2L female; MOF-2LM, MOF-overexpressed trisomy 2L male. (**B**) RT-qPCR analysis of messenger RNA (mRNA) levels of *Ythdc1* in the heads of MSL2 transgenic *Drosophila*. MOF KDF, MOF neural-knockdown female; MOF KDM, MOF neural-knockdown male; MOF OEF, MOF-overexpressed female; MOF OEM, MOF-overexpressed male. Sample size = 3. Student’s *t* test *p<0.05, **p<0.01, ***p<0.001. (**C**) RT-qPCR analysis of mRNA levels of MOF in the heads of *Ythdc1* knockdown *Drosophila*. Ythdc1 KDF, Ythdc1 neural-knockdown female; Ythdc1 KDM, Ythdc1 neural-knockdown male. Sample size = 3. Student’s *t* test *p<0.05, **p<0.01, ***p<0.001. (**D**) Western blot analysis of H4K16Ac in *Drosophila*. (**E,F**) Relative quantification of H4K16Ac in wildtype and *Ythdc1* knockdown adult *Drosophila* based on western blot. Student’s *t* test * Ythdc1 KDF, Ythdc1-knockdown female; Ythdc1 KDM, Ythdc1-knockdown male. Sample size = 5. Student’s *t* test *p<0.05. (**G**) Genome browser example of *Ythdc1* for indicated ChIP-seq data. Signals were displayed as counts per million (CPM) values. The gene architecture was shown at the bottom (only one representative transcript isoform was shown). The yellow shaded area indicates the presence of the H4K16Ac peaks. Figure 8—source data 1.PDF file containing original western blots for Figure 8D, indicating the relevant bands and treatments. Figure 8—source data 2.Original files for western blot analysis displayed in Figure 8D.Figure supplements.

As an important component of the MSL complex, MOF is a histone acetyltransferase that specifically acetylates histone H4 at lysine 16 (H4K16Ac), thereby affecting chromatin structure and functions, and activating transcription (Kind et al., 2008; Conrad et al., 2012). Previous studies have elucidated the regulatory role of m^6^A in histone modifications, and histone 3 lysine 36 trimethylation (H3K36me3) also plays a role in recruiting methyltransferase complexes to deposit m^6^A markers on RNA (Wang et al., 2018; Huang et al., 2019; Li et al., 2020). To investigate the functions of MOF-mediated H4K16Ac on RNA m^6^A modification, we analyzed RNA-seq data from *Drosophila* strains overexpressing MOF (Figure 8A; Figure 7—figure supplement 1J–L). We found obvious changes in the expression of m^6^A regulators in strains overexpressing MOF, with the levels of almost all m^6^A regulators being elevated (Figure 8A). It was also observed that after overexpression of MOF, the increasing trends of m^6^A regulators in females and males were not exactly the same, mostly showing more pronounced increases in females, except for Mettl3 (Ime4) and dMettl14. According to the results of RT-qPCR, the expression of Ythdc1 was decreased in MOF knockdown strains and increased in MOF overexpression strains (Figure 8B; Figure 7—figure supplement 1J); meanwhile, the expression levels of MOF were significantly reduced in *Ythdc1* neural-knockdown strains (Figure 8C). These results were further verified by polytene chromosome immunofluorescence experiments (Figure 8—figure supplement 1E and F). In males, the expression level of MOF was significantly decreased in *Ythdc1* neural-knockdown *Drosophila*; while in *Ythdc1* neural-knockdown females, the expression level of MOF was not significantly changed compared with wildtype females (Figure 8—figure supplement 1E and F). These results suggest that MOF-mediated acetylation modification can have a certain effect on RNA m^6^A methylation in a sexually dimorphic manner, which may be due to the endogenous MSL complex and the imbalance of X chromosome dosage in males.

**Figure 8—figure supplement 1.**
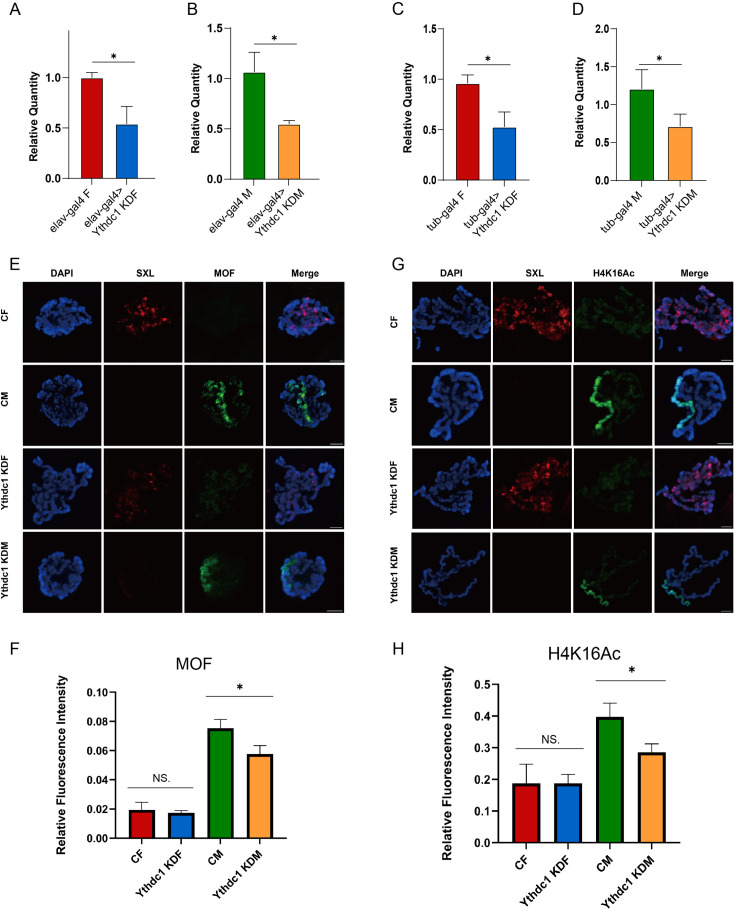
Ythdc1 may regulate H4K16Ac through histone acetyltransferase MOF. (**A,B**) RT-qPCR analysis of messenger RNA (mRNA) levels of Ythdc1 in the heads of *Ythdc1* neural-knockdown female (**A**) and male (**B**). elav-gal4>Ythdc1 KDF, Ythdc1 neural-knockdown female; elav-gal4>Ythdc1 KDM, Ythdc1 neural-knockdown male. Sample size = 3. Student’s *t* test *p<0.05, **p<0.01, ***p<0.001. (**C,D**) RT-qPCR analysis of mRNA levels of Ythdc1 in *Ythdc1* knockdown female (**C**) and male (**D**) adult *Drosophila*. tub-gal4>Ythdc1 KDF, Ythdc1 knockdown female; tub-gal4>Ythdc1 KDM, Ythdc1 knockdown male. Sample size = 3. Student’s *t* test *p<0.05, **p<0.01, ***p<0.001. (**E**) Immunofluorescence of polytene chromosomes showed the signals of SXL (red channel) and MOF (green channel) in the salivary glands of *Drosophila* larvae of different genotypes. Scale bars, 10 μm. (**F**) The levels of MOF in the salivary glands of Ythdc1 knockdown *Drosophila* quantified by immunofluorescence. Sample size > 10. Student’s *t* test *p<0.05, **p<0.01, ***p<0.001.(**G**) Immunofluorescence of polytene chromosomes showed the signals of SXL (red channel) and H4K16Ac (green channel) in the salivary glands of *Drosophila* larvae of different genotypes. Scale bars, 10 μm. (**H**) The levels of H4K16Ac in the salivary glands of Ythdc1 knockdown *Drosophila* quantified by immunofluorescence. Ythdc1 KDF, Ythdc1 knockdown female; Ythdc1 KDM, Ythdc1 knockdown male. Sample size > 10. Student’s *t* test *p<0.05, **p<0.01, ***p<0.001.

**Figure 8—figure supplement 2.**
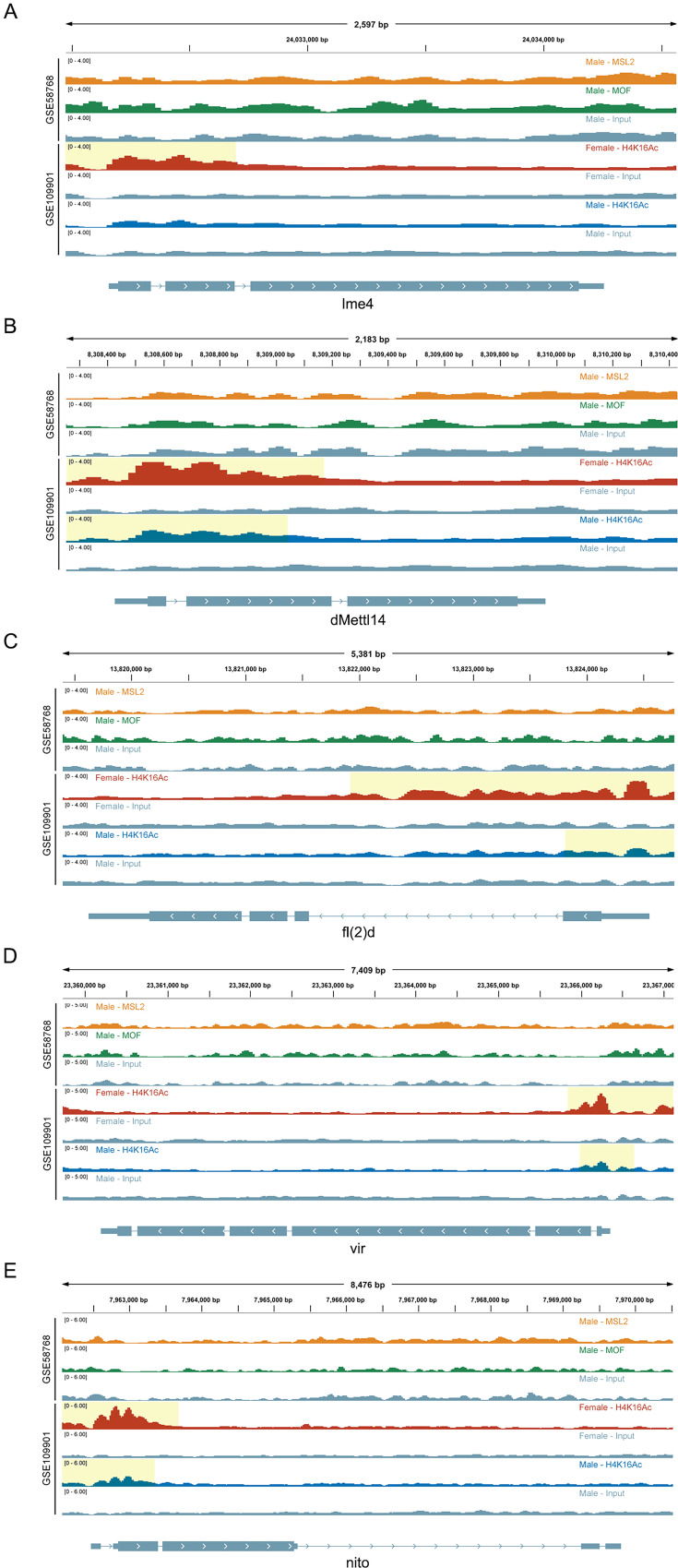
Genome browser example of m^6^A regulator genes for indicated ChIP-seq data. H4K16Ac, MSL2, and MOF ChIP-seq results for gene *Ime4* (**A**), *dMettl14* (**B**), *fl(2)d* (**C**), *vir* (**D**), and *nito* (**E**) are shown, respectively. Signals were displayed as counts per million (CPM) values. The gene architectures were shown at the bottom (only one representative transcript isoform was shown). The yellow shaded area indicates the presence of the H4K16Ac peaks.

Next, we investigated whether the m^6^A reader protein Ythdc1 would also have an effect on the levels of H4K16Ac in *Drosophila*. To this end, we employed western blot analysis to assess the expression patterns of H4K16Ac in *Ythdc1* knockdown *Drosophila* adults (Figure 8D). The subsequent quantitative analysis revealed a significant up-regulation of H4K16Ac in both female and male (Figure 8E and F). Furthermore, to substantiate our findings, we conducted polytene chromosome immunofluorescence in third instar larvae (Figure 8—figure supplement 1G). Quantitative analysis of these assays in *Ythdc1* knockdown *Drosophila* showed that the changes of H4K16Ac levels also showed sexual dimorphism (Figure 8—figure supplement 1H). In males, knockdown of *Ythdc1* led to a decrease in H4K16Ac level; whereas in females, knockdown of *Ythdc1* did not affect the level of H4K16Ac (Figure 8—figure supplement 1H). These observed changes in H4K16Ac were consistent with the pattern of changes in MOF protein in *Ythdc1* knockdown *Drosophila* strains, suggesting that m^6^A modification may affect H4K16Ac levels through the mediation of MOF. Overall, while the alterations in H4K16Ac levels are not uniformly consistent between larvae and adult *Drosophila*, these findings nonetheless demonstrate that the knockdown of *Ythdc1* has a significant impact on the expression levels of H4K16Ac at different stages of development and imply a potential relationship between H4K16Ac with m^6^A modification.

We analyzed two ChIP-seq datasets (GSE109901 and GSE58768) to study whether m^6^A regulator genes (especially *Ythdc1*) are targets of DCC components and H4K16Ac. According to the results, most of the m^6^A regulator genes, including *Ythdc1*, contain H4K16Ac peaks in both sexes, all of which are located in the 5' regions (Figure 8G; Figure 8—figure supplement 2); except that *Ime4* shows sexual dimorphism and only contains H4K16Ac peak in females. On the other hand, analysis of ChIP-seq data of MSL2 and MOF in male *Drosophila* showed that most of MSL2 and MOF peaks were located on the X chromosome (99.1% of MSL2 peaks and 61.6% of MOF peaks), which may be due to the fact that MSL2 and MOF are mostly tethered to the X chromosome by MSL complex under physiological conditions (Bashaw and Baker, 1995; Kelley et al., 1995; Kind et al., 2008; Conrad et al., 2012). Therefore, there is no MSL2 and MOF peak near the m^6^A regulator genes which located on the autosomes (Figure 8H; Figure 8—figure supplement 2). These results showed that there is a direct relationship between m^6^A regulators and H4K16Ac, but there is no evidence that m^6^A regulator genes are direct targets of DCC components. MSL2 and MOF may thereby interact with m^6^A regulators in other ways.

To further study whether unbalanced genomes are involved in the interaction between m^6^A and histone acetylation modification, we also analyzed RNA-seq data from trisomy 2L *Drosophila* strains overexpressing MOF. The data showed that overexpression of MOF in trisomy 2L resulted in significant changes in the expression of m^6^A regulators, with a trend different from that observed in diploids (Figure 8A). These results suggest that genomic imbalance might affect the interaction between MOF and m^6^A regulators to some extent, and the potential mechanisms require further investigation.

## Discussion

As an emerging epigenetic modification, RNA m^6^A methylation has been found to be involved in almost all aspects of RNA fate and metabolism (Gilbert et al., 2016; Yang et al., 2018). RNA m^6^A modification is also closely related to the development of organisms and a variety of human diseases (Barbieri et al., 2017; Zhao et al., 2017; Pinello et al., 2018; Ma et al., 2019; Liu et al., 2021; Shafik et al., 2021). However, the roles of m^6^A methylation in development and gene expression of aneuploidy have not been studied yet. Aneuploid variation is usually more detrimental than changes of the entire chromosome set due to genomic imbalance (Birchler and Veitia, 2007; Birchler and Veitia, 2012). The global changes of gene regulatory networks in unbalanced genomes involves various epigenetic mechanisms, including histone modification, chromatin remodeling, lncRNAs, microRNAs, etc. (Birchler, 2016; Zhang et al., 2021a; Zhang et al., 2023; Shi et al., 2022). This study demonstrated that the expression of m^6^A components was altered under genomic imbalance, leading to dynamic changes in the entire methylome. Potential intermediaries by which m^6^A modification could affect *trans* regulation and achieve dosage compensation in aneuploid *Drosophila* were also investigated, such as dosage-sensitive modifiers, alternative splicing events, and the MSL complex.

Our experiments show that the expression levels of most m^6^A component genes are significantly down-regulated in aneuploid *Drosophila* larvae (Figure 1A; Figure 1—figure supplement 1C). Depletion of m^6^A components interferes with the development of animals and plants, especially leading to impaired self-renewal and differentiation of embryonic stem cells, defects in embryonic development, and even early embryonic lethality (Granadino et al., 1990; Horiuchi et al., 2006; Raffel et al., 2007; Luo et al., 2014; Wang et al., 2014a; Geula et al., 2015). We demonstrated by TSA-FISH that appropriate temporal and spatial specific distributions of the transcripts for m^6^A components are vital during *Drosophila* embryogenesis (Figure 1C–F; Figure 1—figure supplement 2). The abnormal expression of m^6^A-related genes may affect the development of aneuploid embryos.

In previous studies, the abundance of m^6^A modification is usually positively correlated with the number of m^6^A peaks and m^6^A-marked genes (Luo et al., 2014; Zhu et al., 2023). However, our results obviously did not conform to this rule, with higher m^6^A abundance and fewer MeRIP-Seq peaks in aneuploids (Figure 2A–C). This reflects the complexity and heterogeneity of m^6^A modification. We suspect that in aneuploidy many RNAs may be lost that are methylated at a low level in wildtype, and possess a higher proportion of highly methylated RNAs. Analysis of the expression levels at each m^6^A site confirmed our hypothesis (Figure 2D). Thus, this phenomenon represents an imbalance in m^6^A methylation caused by aneuploidy. In addition, it is worth noting that due to the limitation of the larval samples, our detection of the overall abundance of m^6^A in aneuploidy is carried out for total RNA, including all types of RNA such as mRNA, lncRNA, and rRNA, and may be slightly different from detection of mRNA only. However, according to the results of m^6^A abundance detection in *Drosophila* adult heads, the enrichment or non-enrichment of mRNA from total RNA did not make a substantive difference in the results.

The distribution of m^6^A sites on gene features, m^6^A consensus motifs, biotypes of m^6^A-marked genes, enriched functions and pathways of methylated genes in aneuploidies are similar to those in wildtype. We also found that genes highly methylated in all genotypes are enriched for transcription factors (Figure 2I). It is consistent with previous studies suggesting that transcription regulatory genes may be preferentially targeted by m^6^A (Kan et al., 2017).

We also analyzed the characteristics of DMPs and DMP-associated genes in trisomies. By observing the distributions of MeRIP-Seq reads around DMP sites, it can be found that the densities of m^6^A-marked reads on chromosome 2L are relatively higher in trisomy 2L females and males (Figure 3F and G). Meanwhile, metafemales with triple X have a higher density of m^6^A-marked reads on chromosome X (Figure 3H). Previous studies have found that there is dosage compensation for *cis* genes in autosomal and sex chromosome aneuploid *Drosophila*, and the expression of most genes on varied chromosomes approaches diploid levels (Sun et al., 2013b; Sun et al., 2013c). Therefore, the up-regulation of m^6^A levels is not directly caused by the increased number of chromosomes. A recent study found that the selective enrichment of m^6^A methylation may play a role as a transcript degradation signal in dosage compensation in mammals (Rücklé et al., 2023). Therefore, we speculate that changes in m^6^A levels in aneuploid *Drosophila* may affect its dosage compensation and inverse dosage effect by regulating the stability of the transcripts. In addition, more up-regulated DMPs are detected in trisomy 2L males and metafemales, while trisomy 2L females have more down-regulated DMPs (Figure 3A–D). Combined with the fact that the survival rate of trisomy 2L female larvae is relatively lower than that of the other two trisomies, it can be speculated that the regulation of DMPs may affect the survival and development of aneuploid *Drosophila*.

Gene expression in unbalanced genomes is extensively modulated (Sun et al., 2013b; Sun et al., 2013c; Hou et al., 2018; Raznahan et al., 2018; Shi et al., 2021; Yang et al., 2021; Zhang et al., 2021b; San Roman et al., 2023). Previous studies have analyzed the transcriptome data of autosomal and sex chromosome trisomic *Drosophila*, and found that most of the genes on the triple chromosomes were compensated, and the ratios of their expression levels to wildtype were approximately 1; meanwhile, the expression of genes on other chromosomes was close to two-thirds of that of the wildtype, which was called an inverse dosage effect (Sun et al., 2013b; Sun et al., 2013c; Zhang et al., 2021b). We investigated the relationships between genes with different m^6^A methylation status and genes modulated by classical dosage-related effects (Figure 5F–H). The results showed that for aneuploid females, genes methylated in both trisomy and control are significantly enriched in dosage compensated *cis* genes and inverse dosage effect *trans* genes, and genes not methylated at all are enriched in dosage effect genes. However, in aneuploid males, methylated genes are associated with gene dosage effect (Figure 5F–H). We also found that the proportion of 5’UTR m^6^A peaks on dosage compensation and inverse dosage effect genes are increased in aneuploid females (Figure 5I–K). These results provide evidence of sexual dimorphism in the relationships between RNA m^6^A modification and dosage-related effects. m^6^A located in the 5’UTR generally shows higher tissue specificity and richer dynamic changes, and may have unique regulatory functions (Dominissini et al., 2012; Meyer and Jaffrey, 2014; Gilbert et al., 2016). The increased proportion of m^6^A in the 5’UTR of genes with classical dosage-related effects in aneuploidies suggests that m^6^A modification may be involved in the regulation of dosage-dependent genes.

Dosage-related effects of aneuploidy are thought to be caused by dosage-sensitive genes (Shi et al., 2021; Yang et al., 2021). Some dosage-sensitive regulators have been identified in *Drosophila*, and changes in the dosage of individual regulatory genes can mimic the effects of aneuploidy in the whole genome (Birchler et al., 2001; Xie and Birchler, 2012; Zhang et al., 2021b). Most dosage-sensitive regulators are transcription factors, signal transduction components, and chromatin proteins, which have in common being members of macromolecular complexes or having multicomponent interactions (Birchler et al., 2001; Birchler and Veitia, 2007). We studied the m^6^A modification of dosage-dependent genes and their PPI networks in aneuploid *Drosophila* (Figure 5—figure supplement 1). The results showed that there are complex interactions between dosage-sensitive regulators and differentially expressed genes. Among them, most of the DEGs located on the unvaried chromosomes are down-regulated, indicating that the dosage-sensitive regulators mainly have negative effects on *trans* target genes. There are more regulatory genes with up-regulated DMPs than down-regulated DMPs in all three aneuploidies, and many of the regulators with up-regulated methylation are connected with interactors that have down-regulated expression.

We comprehensively analyzed the relationships among RNA m^6^A modification, gene expression levels, and alternative splicing. The data showed that differential m^6^A methylation under genomic imbalance appeared to be more closely associated with differential alternative splicing (Figure 5A–C; Figure 6A–C). This phenomenon is reasonable because the mutation or knockout of *Ime4*, *dMettl14*, *fl(2)d*, and *Ythdc1* has been found to affect a large number of alternative splicing events in *Drosophila*, and *fl(2)d* itself is thought to encode a splicing factor (Penn et al., 2008; Haussmann et al., 2016; Lence et al., 2016). A small set of transcripts are simultaneously differentially m^6^A methylated, differentially expressed, and differentially alternative spliced in aneuploidies, including the transcription factor *su(Hw*), which may be a dosage-sensitive regulator (Figure 6G–H). Besides, m^6^A modification in the 5’UTR regions may play a special role in some differential alternative splicing events (Figure 6—figure supplement 1F–I).

RNA m^6^A modification in *Drosophila* has been shown to be involved in the alternative splicing of *Sxl* (Lence et al., 2017). The deletion of some m^6^A components in females will result in a reduction of female-specific splicing of *Sxl* and an increase of the inclusion of the third exon, along with phenotypic sexual transformation (Haussmann et al., 2016; Lence et al., 2016; Kan et al., 2017). We found that *Sxl* transcripts of aneuploid *Drosophila* are both differentially m^6^A methylated and differentially spliced (Figure 7A and B). Among multiple alternative splicing events, trisomy 2L females have a higher level of the third exon inclusion compared with wildtype females (Figure 7B), which may be related to the differential methylation at 5’UTR of the *Sxl* transcripts.

Some studies proposed that the deletion of RNA m^6^A methyltransferase Ime4 harms the survival of female *Drosophila* because of the insufficient inhibition of *msl-2* caused by decreased *Sxl* levels, which in turn leads to up-regulation of X-linked genes (Haussmann et al., 2016). However, other studies have found that the expression of genes on the X chromosome in females with ectopic expression of MSL2 does not increase twofold, i.e., the MSL complex does not directly mediate dosage compensation, and the global inverse dosage effect caused by the imbalance of sex chromosomes is the basis for dosage compensation (Birchler, 1981; Birchler, 2016; Sun et al., 2013a). Therefore, the lethality of the lack of m^6^A in female *Drosophila* may not be directly caused by up-regulation of X-linked genes through ectopic assembly of MSL complex, and the specific reasons need to be further studied.

The functions of MSL complex in dynamic regulation of global gene expression in aneuploid genomes have been described (Zhang et al., 2021a). Here, we found that MSL2, a structure component of MSL complex, not only affects the expression of m^6^A regulators, but also influences the overall abundance of mRNA m^6^A in *Drosophila* (Figure 7C–F), proving a close relationship between the MSL complex and RNA m^6^A modification. In addition, under the condition of genomic imbalance, the relative expression of m^6^A components was also changed in larvae and embryos of MSL2-overexpressed trisomies (Figure 7G and H). Another component of the MSL complex, MOF, which mediates histone acetylation and transcriptional activation (Kind et al., 2008; Conrad et al., 2012), is also closely associated with the m^6^A reader Ythdc1. Overexpression of MOF increased the expression of Ythdc1 (Figure 8A and B), and in turn, knockdown of *Ythdc1* influenced the expression of MOF and the level of H4K16Ac catalyzed by it (Figure 8C–F; Figure 8—figure supplement 1G and H). It is worth noting that the rationale behind the variable expression levels of H4K16Ac in *Ythdc1* knockdown *Drosophila* across different developmental stages merits further investigation. These results demonstrate complicated interactions between RNA m^6^A methylation and the MSL complex in unbalanced genomes, which may affect the gene expression, sexual dimorphism, development, and survival of aneuploid *Drosophila*.

## Materials and methods

### *Drosophila* stocks and genetic crosses

The *Drosophila* strains mentioned in this study were all maintained and crossed in our laboratory. The crossing methods have been described previously (Zhang et al., 2021a). Trisomy chromosome 2 left arm (2L) female and male third instar larvae were obtained from the cross of *y; C(2L)dp; F(2R) bw* females and Canton S males. The metafemale larvae were obtained from the cross of *C(1)DX, ywf/winscy* females and Canton S males. Aneuploidies overexpressing MSL2 were generated from the crosses of *Drosophila* with compound chromosomes and MSL2 homozygotes. The MSL2 transgene strain was constructed and validated in a previous study (Sun et al., 2013a). All *Drosophila* strains were cultured on cornmeal dextrose medium at 25°C. Genes and chromosomal balancers are described in Flybase (https://flybase.org/).

### RNA m^6^A methylation quantification

Total RNA was extracted from *Drosophila* larvae using TRIzol Reagent (Invitrogen) and mRNA was isolated using the Dynabeads mRNA purification kit (Invitrogen, 61006) to detect the abundance of m^6^A. The relative quantification of m^6^A methylation was performed using EpiQuik m^6^A RNA Methylation Quantification Kit (Colorimetric) (Epigentek, NY, USA, Cat # P-9005). Specifically, 80 µl of Binding Solution was first added to each well of the plate, and 200 ng of total RNA or mRNA samples, 2 µl of Negative Control, or 2 µl of diluted Positive Control were added to the designed wells. Subsequently, the plate was incubated at 37°C for 90 min. After washing with Wash Buffer, 50 µl of Capture Antibody, 50 µl of Detection Antibody, and 50 µl of Enhancer Solution were added to each well in order, and wells were emptied before adding a new solution each time. Finally, 100 µl of Developer Solution and Stop Solution were added to each well away from light, and the absorbance was read on a microplate reader at a wavelength of 450 nm.

### m^6^A methylated RNA immunoprecipitation sequencing

The MeRIP-Seq service was provided by CloudSeq Biotech Inc (Shanghai, China), and this technology was developed on the basis of published experimental methods (Meyer et al., 2012). In brief, *Drosophila* larvae of five genotypes, each with two biological replicates, were collected for sequencing. Ribosomal RNAs were removed from total RNA using Ribo-Zero rRNA Removal Kits (Illumina, USA). Immunoprecipitation of m^6^A RNA was performed using GenSeq m^6^A RNA IP kit (GenSeq, Shanghai, China). The NEBNext Ultra II Directional RNA Library Prep kit (New England Biolabs, USA) was used for RNA-seq library construction. High-throughput sequencing was performed using Illumina NovaSeq 6000 sequencers with the paired-end 150 bp protocol.

### Analysis of MeRIP-Seq data

The raw sequencing data was first filtered by Trim Galore (version 0.6.10) (https://www.bioinformatics.babraham.ac.uk/projects/trim_galore/) to remove adapters and low-quality reads. The quality of the data was then assessed by FastQC (version 0.12.1) (https://www.bioinformatics.babraham.ac.uk/projects/fastqc/). Subsequently, clean reads were aligned to the *Drosophila* reference genome (Drosophila_melanogaster.BDGP6.32.dna.toplevel.fa, downloaded from the Ensembl database) using HISAT2 (version 2.2.1) (Kim et al., 2019). Next, The R package exomePeak2 (version 1.10.0) (Meng et al., 2013) was used for m^6^A peak calling (the screening criteria were log2FC≥1, RPM.IP≥0.5, and score≥5). Motif analysis was performed using HOMER (version 4.11) (Heinz et al., 2010). DMPs were analyzed by the R package DiffBind (version 3.8.4) (Stark and Brown, 2013), which employs the DESeq2 (Love et al., 2014) method. m^6^A peaks with a p-value of 0.1 or less were considered as DMPs. ChIPseeker (version 1.34.1) (Yu et al., 2015) was used to annotate the peaks, and plotted some of the figures. The profiles and heatmaps of MeRIP-Seq reads around DMPs were generated by deepTools (version 3.5.4) (Ramírez et al., 2014). The signal distribution on the genome was visualized using the Integrative Genomics Viewer (IGV) software (version 2.12.0) (Thorvaldsdóttir et al., 2013).

### RNA-seq data and analysis

The RNA-seq data of aneuploid *Drosophila* used in this article were generated in a previous study (Zhang et al., 2023) and can be downloaded from the Gene Expression Omnibus (GEO) database (GSE233534). Genome mapping of sequencing data and gene expression quantification were carried out through HISAT2-StringTie pipeline (Pertea et al., 2015; Kim et al., 2019). Differential alternative splicing events were identified by rMATS (version 4.2.0) program (Shen et al., 2014) (with the parameters of -t paired --readLength 150 --cstat 0.0001 --libType fr-firststrand). The method of generating the ratio distributions of gene expression changes was as described previously (Zhang et al., 2022). The plots were generated using ggplot2 (version 3.4.0) (Villanueva and Chen, 2019) in the R program (version 4.2.2). Differential expression analysis was performed by DESeq2 (version 1.38.2) (Love et al., 2014), and the threshold was set to adjusted p-value≤0.05. The functional enrichment analysis was performed by ClusterProfiler (version 4.6.0) (Wu et al., 2021) based on org.Dm.eg.db (version 3.16.0) from Bioconductor (https://www.bioconductor.org/). The KEGG pathway data was obtained from the network (https://www.kegg.jp/). The list of transcription factors of *Drosophila* was downloaded from AnimalTFDB (Hu et al., 2019). PPI relationships were obtained from the STRING database (Szklarczyk et al., 2019).

### Real-time quantitative PCR

Total RNA of *Drosophila* whole larvae, larval brains, or adult heads was extracted using TRIzol Reagent (Invitrogen), and reverse transcription was done with *TransScript* one-step gDNA Removal and cDNA Synthesis SuperMix (TransGen Biotech). The sequences of primers designed for RT-qPCR were listed in Figure 1—figure supplement 1A. The usability of these primers was verified by agarose gel electrophoresis (Figure 1—figure supplement 1B). The real-time PCR was performed with *TransStart* Tip Green qPCR SuperMix (+Dye II) (TransGen Biotech) using ABI QuantStudio 6 Flex Real-Time PCR System. Relative quantification of gene expression was determined using the 2^-∆∆Ct^ method.

### FISH of *Drosophila* embryos

Collection of *Drosophila* embryos and FISH were performed as previously described (Jandura et al., 2017; Zhang et al., 2021a). The probe primers containing flanking T7 promoter elements were listed in Figure 1—figure supplement 1D. After PCR amplification and in vitro transcription, their products were examined by agarose gel electrophoresis (Figure 1—figure supplement 1E). Digoxygenin (DIG)-labeled antisense RNA probes were hybridized to transcripts of interest, and then detected using a succession of anti-DIG antibody conjugated to biotin, streptavidin conjugated to horseradish peroxidase (HRP) and fluorescently conjugated tyramide. This hierarchical process can greatly enhance the probe signals and is referred to as a TSA system. All images were acquired with a Zeiss LSM880 laser confocal fluorescence microscope using ZEN software. The same probes for relative fluorescence intensity analysis were photographed using the same parameters. Fluorescence images were processed and analyzed using Fiji (version 1.53c) (Schindelin et al., 2012).

### Polytene chromosomes immunostaining

Salivary gland chromosomes immunostaining was performed as previously described (Zhang et al., 2021a). Briefly speaking, the salivary glands from third-instar larvae were first fixed in 3.7% formaldehyde for 1 min, and then dissociated with 50% acetic acid for 5 min. Polytene chromosomes were treated with the following primary antibodies at a dilution of 1:100: anti-SXL (Developmental Studies Hybridoma Bank, M18-s), anti-MSL2 (Santa Cruz, sc-32459), anti-MOF (Santa Cruz, sc-22351), and anti-H4K16Ac (EMD Millipore, 07-329). Finally, fluorescence-conjugated secondary antibodies (Alexa Fluor 488 and Alexa Fluor 594, Jackson ImmunoResearch) were used for detection.

### Histone extraction and western blotting

*Drosophila* adults were ground and lysed in Triton Extraction Buffer (PBS at pH 7.4 with 0.5% Triton X-100, 0.5 mM phenymethylsulfonyl fluoride, and 0.02% sodium butyrate) and histones were acid-extracted in 0.2 N HCl overnight. Acid-extracted histones were then run on a 12% Tris-Glycine gel and blotted onto a PVDF membrane. Antibodies for Histone H3 (NB500-171, Novus) and H4K16Ac (07-329, Sigma) were all incubated at a dilution of 1:1000 in 5% skim milk powder solution. Westerns blots were imaged and protein levels quantified using the ImageJ software.

## Data Availability

Sequencing data generated in this study have been deposited in GEO under accession number GSE253401. The following dataset was generated: ZhangS LiuX WangR WangJ ZhangL SunL 2024Dynamics and regulatory roles of RNA m 6 A methylation in unbalanced genomesNCBI Gene Expression OmnibusGSE25340110.7554/eLife.100144PMC1175941039853090 The following previously published datasets were used: ZhangS WangR ZhuX ZhangL LiuX SunL 2024Characteristics and expression of lncRNA in *Drosophila* aneuploidyNCBI Gene Expression OmnibusGSE23353410.1016/j.isci.2023.108494PMC1073089238125016 RenschlerG RichardG ValsecchiCIK ToscanoS 2018Facultative dosage compensation of developmental genes on autosomes in *Drosophila* and mammalsNCBI Gene Expression OmnibusGSE10990110.1038/s41467-018-05642-2PMC612890230194291 FigueiredoML KimM PhilipP AllgardssonA StenbergP LarssonJ 2014Non-coding roX RNAs prevent the binding of the MSL-complex to heterochromatic regionsNCBI Gene Expression OmnibusGSE5876810.1371/journal.pgen.1004865PMC426346525501352

## Additional files

MDAR checklist

## Appendix 1

**Appendix 1—key resources table:**

Reagent type (species) or resource	Designation	Source or reference	Identifiers	Additional information
Antibody	anti-SXL (Mouse monoclonal)	Developmental Studies Hybridoma Bank	Cat# M18-s, RRID:AB_528464	IF(1:100)
Antibody	anti-MSL2 (Goat monoclonal)	Santa Cruz	Cat# sc-32459, RRID:AB_672213	IF(1:100)
Antibody	anti-MOF (Goat monoclonal)	Santa Cruz	Cat# sc-22351, RRID:AB_670132	IF(1:100)
Antibody	anti-H3 (Rabbit polyclonal)	Novus	Cat# NB500-171, RRID:AB_10001790	WB(1:1000)
Antibody	anti-H4K16Ac (Rabbit monoclonal)	EMD Millipore	Cat# 07-329, RRID:AB_310525	IF(1:100) WB(1:1000)
Antibody	anti-Digoxigenin (Mouse monoclonal)	Jackson Immuno Research	Cat# 200-062-156, RRID:AB_233901	TSA-FISH(1:400)
Antibody	Streptavidin-HRP (Rabbit polyclonal)	Invitrogen	Cat# S991	TSA-FISH(1:1000)
Sequence-based reagent	Ime4-L	This paper	PCR primers	CAAGTACGTGCACTATGAGG
Sequence-based reagent	Ime4-R	This paper	PCR primers	GTCATGTCCAAGAAGCGTAG
Sequence-based reagent	dMettl14-L	This paper	PCR primers	GCCTCTTCCTCCAAGAAAAC
Sequence-based reagent	dMettl14-R	This paper	PCR primers	CCTCAACTTGGGATACTCCT
Sequence-based reagent	fl(2)d-L	This paper	PCR primers	CCTGGACGTTATTTCCTACT
Sequence-based reagent	fl(2)d-R	This paper	PCR primers	TAAGCTAGAGACCATTCACG
Sequence-based reagent	vir-L	This paper	PCR primers	GTACATGAAACCCTTAGAGGC
Sequence-based reagent	vir-R	This paper	PCR primers	CTTGCTTATGGAGAGATAGCG
Sequence-based reagent	nito-L	This paper	PCR primers	GCCAGTACGGTTCCAGATGT
Sequence-based reagent	nito-R	This paper	PCR primers	CCGTCCGTCAAATGAAACTT
Sequence-based reagent	Ythdc1-L	This paper	PCR primers	GGTCGTGATTTGATCCTCTG
Sequence-based reagent	Ythdc1-R	This paper	PCR primers	TCGAACTCACTCCCATACTC
Sequence-based reagent	tubulin-F	This paper	PCR primers	AGCTCAGCACCCTCTGTGTAAT
Sequence-based reagent	tubulin-R	This paper	PCR primers	AGCTGGAGCGCATCAATGTGTA
Sequence-based reagent	Ime4-F-T3	This paper	FISH primers	TGTTGGGAAATCACTCCCAATTAAGAGAAGTTTAAGTCCCACGG
Sequence-based reagent	Ime4-R-T7	This paper	FISH primers	GTAATACGACTCACTATAGGGAGACCACCATCTGGATAACGCTTCTGG
Sequence-based reagent	dMettl14-F-T3	This paper	FISH primers	TGTTGGGAAATCACTCCCAATTAACCAATCCGCACAATGACTAC
Sequence-based reagent	dMettl14-R-T7	This paper	FISH primers	GTAATACGACTCACTATAGGGAGACCACTAGCCAACCTGGTCGAATAC
Sequence-based reagent	fl(2)d-F-T3	This paper	FISH primers	TGTTGGGAAATCACTCCCAATTAACTAGAGACCATTCACGAGGA
Sequence-based reagent	fl(2)d-R-T7	This paper	FISH primers	GTAATACGACTCACTATAGGGAGACCACATTATGTATGTCTACGCCGC
Sequence-based reagent	vir-F-T3	This paper	FISH primers	TGTTGGGAAATCACTCCCAATTAACGAAATGTCGTACAAGGTGC
Sequence-based reagent	vir-R-T7	This paper	FISH primers	GTAATACGACTCACTATAGGGAGACCACGATCTCCGGGAAAAGTGGTT
Sequence-based reagent	nito-F-T3	This paper	FISH primers	TGTTGGGAAATCACTCCCAATTAACTCCGATTGATCCCTACGAT
Sequence-based reagent	nito-R-T7	This paper	FISH primers	GTAATACGACTCACTATAGGGAGACCACGTATCTCCGTCCGTCAAATG
Sequence-based reagent	Ythdc1-F-T3	This paper	FISH primers	TGTTGGGAAATCACTCCCAATTAACGAATCGAATGGTGGAGACT
Sequence-based reagent	Ythdc1-R-T7	This paper	FISH primers	GTAATACGACTCACTATAGGGAGACCACCCGTGTGTCTCGGAATAGGT
Commercial assay or kit	m6A RNA Methylation Quantification Kit (Colorimetric)	EpiQuik	P-9005–96	
Commercial assay or kit	2×EasyTaq PCR Super Mix (+Dye)	TransGen Biotech	AS111-12	
Commercial assay or kit	TransTaq-T DNA Polymerase	TransGen Biotech	AP122	
Commercial assay or kit	TransScript one-step gDNA Removal and cDNA Synthesis SuperMix	TransGen Biotech	AT311-03	
Commercial assay or kit	TransStart Tip Green qPCR SuperMix (+Dye II)	TransGen Biotech	AQ142-24	
Chemical compound, drug	Cyanine 3 Tyramide	Akoya Biosciences	FP1046	
Chemical compound, drug	Proteinase K	Sigma	H4784	
Chemical compound, drug	Heparin sodium salt	Sigma	P2308	
Chemical compound, drug	Salmon sperm single-stranded DNA	Sigma	D9156	
Chemical compound, drug	Digoxigenin-11-UTP	Sigma (Roche Diagnostics)	11209256910	
Chemical compound, drug	Ribonucleoside Triphosphate Set (NTP)	Sigma (Roche Diagnostics)	11277057001	
Chemical compound, drug	Donkey Serum	Solarbio	N/A	
Chemical compound, drug	5×Transcription buffer	Thermo Scientific	N/A	
Chemical compound, drug	T7 RNA polymerase	Thermo Scientific	EP0111	
Software, algorithm	R v4.2.2	http://www.R-project.org		
Software, algorithm	trim-galore v0.6.10	https://www.bioinformatics.babraham.ac.uk/projects/trim_galore/		
Software, algorithm	FastQC v0.12.1	https://www.bioinformatics.babraham.ac.uk/projects/fastqc/		
Software, algorithm	HISAT2 v2.2.1	Kim et al., 2019		
Software, algorithm	StringTie v2.1.5	Pertea et al., 2015		
Software, algorithm	exomePeak2 v1.10.0	Meng et al., 2013		
Software, algorithm	HOMER v4.11	Heinz et al., 2010		
Software, algorithm	DiffBind v3.8.4	Stark and Brown, 2013		
Software, algorithm	ChIPseeker v1.34.1	Yu et al., 2015		
Software, algorithm	deepTools v3.5.4	Ramírez et al., 2014		
Software, algorithm	rMATS v4.2.0	Shen et al., 2014		
Software, algorithm	ggplot2 v3.4.0	Villanueva and Chen, 2019		
Software, algorithm	clusterProfiler v4.6.0	Wu et al., 2021		
Software, algorithm	org.Dm.eg.db v3.16.0	https://www.bioconductor.org/		
Software, algorithm	DESeq2 v1.38.2	Love et al., 2014		
Software, algorithm	Integrative Genomics Viewer v2.12.0	Thorvaldsdóttir et al., 2013		
Software, algorithm	Fiji v1.53c	Schindelin et al., 2012		
Software, algorithm	AnimalTFDB	Hu et al., 2019		
Other	DAPI stain	Sigma	28718-90-3	(1 µg/mL)

## References

[bib1] Barbieri I, Tzelepis K, Pandolfini L, Shi J, Millán-Zambrano G, Robson SC, Aspris D, Migliori V, Bannister AJ, Han N, De Braekeleer E, Ponstingl H, Hendrick A, Vakoc CR, Vassiliou GS, Kouzarides T (2017). Promoter-bound METTL3 maintains myeloid leukaemia by m^6^A-dependent translation control. Nature.

[bib2] Bashaw GJ, Baker BS (1995). The msl-2 dosage compensation gene of *Drosophila* encodes a putative DNA-binding protein whose expression is sex specifically regulated by sex-lethal. Development.

[bib3] Ben-David U, Amon A (2020). Context is everything: aneuploidy in cancer. Nature Reviews. Genetics.

[bib4] Birchler JA (1981). The genetic basis of dosage compensation of alcohol dehydrogenase-1 in maize. Genetics.

[bib5] Birchler JA, Bhadra U, Bhadra MP, Auger DL (2001). Dosage-dependent gene regulation in multicellular eukaryotes: implications for dosage compensation, aneuploid syndromes, and quantitative traits. Developmental Biology.

[bib6] Birchler JA, Veitia RA (2007). The gene balance hypothesis: from classical genetics to modern genomics. The Plant Cell.

[bib7] Birchler JA, Veitia RA (2012). Gene balance hypothesis: connecting issues of dosage sensitivity across biological disciplines. PNAS.

[bib8] Birchler JA (2016). Parallel universes for models of X chromosome dosage compensation in *Drosophila*: a review. Cytogenetic and Genome Research.

[bib9] Birchler JA, Veitia RA (2021). One hundred years of gene balance: how stoichiometric issues affect gene expression, genome evolution, and quantitative traits. Cytogenetic and Genome Research.

[bib10] Chiarle R (2021). Solving the chromosome puzzle of aneuploidy in cancer. Genes & Development.

[bib11] Conrad T, Cavalli FMG, Holz H, Hallacli E, Kind J, Ilik I, Vaquerizas JM, Luscombe NM, Akhtar A (2012). The MOF chromobarrel domain controls genome-wide H4K16 acetylation and spreading of the MSL complex. Developmental Cell.

[bib12] Disteche CM (2016). Dosage compensation of the sex chromosomes and autosomes. Seminars in Cell & Developmental Biology.

[bib13] Dominissini D, Moshitch-Moshkovitz S, Schwartz S, Salmon-Divon M, Ungar L, Osenberg S, Cesarkas K, Jacob-Hirsch J, Amariglio N, Kupiec M, Sorek R, Rechavi G (2012). Topology of the human and mouse m6A RNA methylomes revealed by m6A-seq. Nature.

[bib14] Geula S, Moshitch-Moshkovitz S, Dominissini D, Mansour AA, Kol N, Salmon-Divon M, Hershkovitz V, Peer E, Mor N, Manor YS, Ben-Haim MS, Eyal E, Yunger S, Pinto Y, Jaitin DA, Viukov S, Rais Y, Krupalnik V, Chomsky E, Hanna JH (2015). Stem cells: m6A mRNA methylation facilitates resolution of naive pluripotency toward differentiation. Science.

[bib15] Gilbert WV, Bell TA, Schaening C (2016). Messenger RNA modifications: form, distribution, and function. Science.

[bib16] Granadino B, Campuzano S, Sánchez L (1990). The *Drosophila melanogaster* fl(2)d gene is needed for the female-specific splicing of sex-lethal RNA. The EMBO Journal.

[bib17] Gurudatta BV, Ramos E, Corces VG (2012). The BEAF insulator regulates genes involved in cell polarity and neoplastic growth. Developmental Biology.

[bib18] Haussmann IU, Bodi Z, Sanchez-Moran E, Mongan NP, Archer N, Fray RG, Soller M (2016). m^6^A potentiates Sxl alternative pre-mRNA splicing for robust *Drosophila* sex determination. Nature.

[bib19] Heinz S, Benner C, Spann N, Bertolino E, Lin YC, Laslo P, Cheng JX, Murre C, Singh H, Glass CK (2010). Simple combinations of lineage-determining transcription factors prime cis-regulatory elements required for macrophage and B cell identities. Molecular Cell.

[bib20] Hongay CF, Orr-Weaver TL (2011). *Drosophila* inducer of MEiosis 4 (IME4) is required for notch signaling during oogenesis. PNAS.

[bib21] Horiuchi K, Umetani M, Minami T, Okayama H, Takada S, Yamamoto M, Aburatani H, Reid PC, Housman DE, Hamakubo T, Kodama T (2006). Wilms’ tumor 1-associating protein regulates G2/M transition through stabilization of cyclin A2 mRNA. PNAS.

[bib22] Hou J, Shi X, Chen C, Islam MS, Johnson AF, Kanno T, Huettel B, Yen MR, Hsu FM, Ji T, Chen PY, Matzke M, Matzke AJM, Cheng J, Birchler JA (2018). Global impacts of chromosomal imbalance on gene expression in *Arabidopsis* and other taxa. PNAS.

[bib23] Hu H, Miao YR, Jia LH, Yu QY, Zhang Q, Guo AY (2019). AnimalTFDB 3.0: a comprehensive resource for annotation and prediction of animal transcription factors. Nucleic Acids Research.

[bib24] Huang H, Weng H, Zhou K, Wu T, Zhao BS, Sun M, Chen Z, Deng X, Xiao G, Auer F, Klemm L, Wu H, Zuo Z, Qin X, Dong Y, Zhou Y, Qin H, Tao S, Du J, Liu J, Lu Z, Yin H, Mesquita A, Yuan CL, Hu YC, Sun W, Su R, Dong L, Shen C, Li C, Qing Y, Jiang X, Wu X, Sun M, Guan JL, Qu L, Wei M, Müschen M, Huang G, He C, Yang J, Chen J (2019). Histone H3 trimethylation at lysine 36 guides m6A RNA modification co-transcriptionally. Nature.

[bib25] Huang AC, Olson SB, Maslen CL (2021). A review of recent developments in turner syndrome research. Journal of Cardiovascular Development and Disease.

[bib26] Jaenisch R, Bird A (2003). Epigenetic regulation of gene expression: how the genome integrates intrinsic and environmental signals. Nature Genetics.

[bib27] Jandura A, Hu J, Wilk R, Krause HM (2017). High resolution fluorescent in situ hybridization in *Drosophila* embryos and tissues using tyramide signal amplification. Journal of Visualized Experiments.

[bib28] Jordan W, Rieder LE, Larschan E (2019). Diverse genome topologies characterize dosage compensation across species. Trends in Genetics.

[bib29] Kan L, Grozhik AV, Vedanayagam J, Patil DP, Pang N, Lim KS, Huang YC, Joseph B, Lin CJ, Despic V, Guo J, Yan D, Kondo S, Deng WM, Dedon PC, Jaffrey SR, Lai EC (2017). The m6A pathway facilitates sex determination in *Drosophila*. Nature Communications.

[bib30] Kelley RL, Solovyeva I, Lyman LM, Richman R, Solovyev V, Kuroda MI (1995). Expression of msl-2 causes assembly of dosage compensation regulators on the X chromosomes and female lethality in *Drosophila*. Cell.

[bib31] Kim D, Paggi JM, Park C, Bennett C, Salzberg SL (2019). Graph-based genome alignment and genotyping with HISAT2 and HISAT-genotype. Nature Biotechnology.

[bib32] Kind J, Vaquerizas JM, Gebhardt P, Gentzel M, Luscombe NM, Bertone P, Akhtar A (2008). Genome-wide analysis reveals MOF as a key regulator of dosage compensation and gene expression in *Drosophila*. Cell.

[bib33] Lécuyer E, Yoshida H, Parthasarathy N, Alm C, Babak T, Cerovina T, Hughes TR, Tomancak P, Krause HM (2007). Global analysis of mRNA localization reveals a prominent role in organizing cellular architecture and function. Cell.

[bib34] Lee M, Kim B, Kim VN (2014). Emerging roles of RNA modification: m(6)A and U-tail. Cell.

[bib35] Lence T, Akhtar J, Bayer M, Schmid K, Spindler L, Ho CH, Kreim N, Andrade-Navarro MA, Poeck B, Helm M, Roignant JY (2016). m6A modulates neuronal functions and sex determination in *Drosophila*. Nature.

[bib36] Lence T, Soller M, Roignant JY (2017). A fly view on the roles and mechanisms of the m(6)A mRNA modification and its players. RNA Biology.

[bib37] Letourneau A, Santoni FA, Bonilla X, Sailani MR, Gonzalez D, Kind J, Chevalier C, Thurman R, Sandstrom RS, Hibaoui Y, Garieri M, Popadin K, Falconnet E, Gagnebin M, Gehrig C, Vannier A, Guipponi M, Farinelli L, Robyr D, Migliavacca E, Borel C, Deutsch S, Feki A, Stamatoyannopoulos JA, Herault Y, van Steensel B, Guigo R, Antonarakis SE (2014). Domains of genome-wide gene expression dysregulation in down’s syndrome. Nature.

[bib38] Li Y, Xia L, Tan K, Ye X, Zuo Z, Li M, Xiao R, Wang Z, Liu X, Deng M, Cui J, Yang M, Luo Q, Liu S, Cao X, Zhu H, Liu T, Hu J, Shi J, Xiao S, Xia L (2020). N6-methyladenosine co-transcriptionally directs the demethylation of histone H3K9me2. Nature Genetics.

[bib39] Liu J, Gao M, He J, Wu K, Lin S, Jin L, Chen Y, Liu H, Shi J, Wang X, Chang L, Lin Y, Zhao YL, Zhang X, Zhang M, Luo GZ, Wu G, Pei D, Wang J, Bao X, Chen J (2021). The RNA m6A reader YTHDC1 silences retrotransposons and guards ES cell identity. Nature.

[bib40] Love MI, Huber W, Anders S (2014). Moderated estimation of fold change and dispersion for RNA-seq data with DESeq2. Genome Biology.

[bib41] Lucchesi JC (2018). Transcriptional modulation of entire chromosomes: dosage compensation. Journal of Genetics.

[bib42] Luo GZ, MacQueen A, Zheng G, Duan H, Dore LC, Lu Z, Liu J, Chen K, Jia G, Bergelson J, He C (2014). Unique features of the m6A methylome in *Arabidopsis thaliana*. Nature Communications.

[bib43] Ma S, Chen C, Ji X, Liu J, Zhou Q, Wang G, Yuan W, Kan Q, Sun Z (2019). The interplay between m6A RNA methylation and noncoding RNA in cancer. Journal of Hematology & Oncology.

[bib44] Meng J, Cui XD, Rao MK, Chen YD, Huang YF (2013). Exome-based analysis for RNA epigenome sequencing data. Bioinformatics.

[bib45] Meyer KD, Saletore Y, Zumbo P, Elemento O, Mason CE, Jaffrey SR (2012). Comprehensive analysis of mRNA methylation reveals enrichment in 3’ UTRs and near stop codons. Cell.

[bib46] Meyer KD, Jaffrey SR (2014). The dynamic epitranscriptome: N6-methyladenosine and gene expression control. Nature Reviews. Molecular Cell Biology.

[bib47] Meyer KD, Patil DP, Zhou J, Zinoviev A, Skabkin MA, Elemento O, Pestova TV, Qian SB, Jaffrey SR (2015). 5’ UTR m(6)A promotes cap-independent translation. Cell.

[bib48] Orr B, Godek KM, Compton D (2015). Aneuploidy. Current Biology.

[bib49] Park SW, Oh H, Lin YR, Park Y (2010). MSL cis-spreading from roX gene up-regulates the neighboring genes. Biochemical and Biophysical Research Communications.

[bib50] Patil DP, Chen CK, Pickering BF, Chow A, Jackson C, Guttman M, Jaffrey SR (2016). m(6)A RNA methylation promotes XIST-mediated transcriptional repression. Nature.

[bib51] Penn JK, Graham P, Deshpande G, Calhoun G, Chaouki AS, Salz HK, Schedl P (2008). Functioning of the *Drosophila* wilms’-tumor-1-associated protein homolog, Fl(2)d, in sex-lethal-dependent alternative splicing. Genetics.

[bib52] Pertea M, Pertea GM, Antonescu CM, Chang TC, Mendell JT, Salzberg SL (2015). Stringtie enables improved reconstruction of a transcriptome from RNA-seq reads. Nature Biotechnology.

[bib53] Pinello N, Sun S, Wong JJ-L (2018). Aberrant expression of enzymes regulating m(6)A mRNA methylation: implication in cancer. Cancer Biology & Medicine.

[bib54] Prestel M, Feller C, Becker PB (2010). Dosage compensation and the global re-balancing of aneuploid genomes. Genome Biology.

[bib55] Rabinow L, Birchler JA (1989). A dosage-sensitive modifier of retrotransposon-induced alleles of the *Drosophila* white locus. The EMBO Journal.

[bib56] Raffel GD, Mercher T, Shigematsu H, Williams IR, Cullen DE, Akashi K, Bernard OA, Gilliland DG (2007). Ott1(Rbm15) has pleiotropic roles in hematopoietic development. PNAS.

[bib57] Ramírez F, Dündar F, Diehl S, Grüning BA, Manke T (2014). deepTools: a flexible platform for exploring deep-sequencing data. Nucleic Acids Research.

[bib58] Raznahan A, Parikshak NN, Chandran V, Blumenthal JD, Clasen LS, Alexander-Bloch AF, Zinn AR, Wangsa D, Wise J, Murphy DGM, Bolton PF, Ried T, Ross J, Giedd JN, Geschwind DH (2018). Sex-chromosome dosage effects on gene expression in humans. PNAS.

[bib59] Roseman RR, Pirrotta V, Geyer PK (1993). The su(Hw) protein insulates expression of the *Drosophila melanogaster* white gene from chromosomal position-effects. The EMBO Journal.

[bib60] Roundtree IA, Evans ME, Pan T, He C (2017a). Dynamic RNA modifications in gene expression regulation. Cell.

[bib61] Roundtree IA, Luo GZ, Zhang ZJ, Wang X, Zhou T, Cui YQ, Sha JH, Huang XX, Guerrero L, Xie P, He E, Shen B, He C (2017b). YTHDC1 mediates nuclear export of N6-methyladenosine methylated mRNAs. eLife.

[bib62] Rücklé C, Körtel N, Basilicata MF, Busch A, Zhou Y, Hoch-Kraft P, Tretow K, Kielisch F, Bertin M, Pradhan M, Musheev M, Schweiger S, Niehrs C, Rausch O, Zarnack K, Keller Valsecchi CI, König J (2023). RNA stability controlled by m(6)A methylation contributes to X-to-autosome dosage compensation in mammals. Nature Structural & Molecular Biology.

[bib63] Sanchez-Pavon E, Mendoza H, Garcia-Ferreyra J (2021). Trisomy 21 and assisted reproductive technologies: a review. JBRA Assist Reprod.

[bib64] San Roman AK, Godfrey AK, Skaletsky H, Bellott DW, Groff AF, Harris HL, Blanton LV, Hughes JF, Brown L, Phou S, Buscetta A, Kruszka P, Banks N, Dutra A, Pak E, Lasutschinkow PC, Keen C, Davis SM, Tartaglia NR, Page DC (2023). The human inactive X chromosome modulates expression of the active X chromosome. Cell Genom.

[bib65] Schindelin J, Arganda-Carreras I, Frise E, Kaynig V, Longair M, Pietzsch T, Preibisch S, Rueden C, Saalfeld S, Schmid B, Tinevez JY, White DJ, Hartenstein V, Eliceiri K, Tomancak P, Cardona A (2012). Fiji: an open-source platform for biological-image analysis. Nature Methods.

[bib66] Shafik AM, Zhang F, Guo Z, Dai Q, Pajdzik K, Li Y, Kang Y, Yao B, Wu H, He C, Allen EG, Duan R, Jin P (2021). N6-methyladenosine dynamics in neurodevelopment and aging, and its potential role in Alzheimer’s disease. Genome Biology.

[bib67] Shen S, Park JW, Lu Z, Lin L, Henry MD, Wu YN, Zhou Q, Xing Y (2014). rMATS: robust and flexible detection of differential alternative splicing from replicate RNA-Seq data. PNAS.

[bib68] Shi X, Yang H, Chen C, Hou J, Hanson KM, Albert PS, Ji T, Cheng J, Birchler JA (2021). Genomic imbalance determines positive and negative modulation of gene expression in diploid maize. The Plant Cell.

[bib69] Shi X, Yang H, Chen C, Hou J, Ji T, Cheng J, Birchler JA (2022). Dosage-sensitive miRNAs trigger modulation of gene expression during genomic imbalance in maize. Nature Communications.

[bib70] Stark R, Brown G, Stark R, Brown G (2013). Bioconductor Package.

[bib71] Sun L, Fernandez HR, Donohue RC, Li J, Cheng J, Birchler JA (2013a). Male-specific lethal complex in *Drosophila* counteracts histone acetylation and does not mediate dosage compensation. PNAS.

[bib72] Sun L, Johnson AF, Donohue RC, Li J, Cheng J, Birchler JA (2013b). Dosage compensation and inverse effects in triple X metafemales of *Drosophila*. PNAS.

[bib73] Sun L, Johnson AF, Li J, Lambdin AS, Cheng J, Birchler JA (2013c). Differential effect of aneuploidy on the X chromosome and genes with sex-biased expression in *Drosophila*. PNAS.

[bib74] Szklarczyk D, Gable AL, Lyon D, Junge A, Wyder S, Huerta-Cepas J, Simonovic M, Doncheva NT, Morris JH, Bork P, Jensen LJ, Mering von C (2019). STRING v11: protein-protein association networks with increased coverage, supporting functional discovery in genome-wide experimental datasets. Nucleic Acids Research.

[bib75] Thorvaldsdóttir H, Robinson JT, Mesirov JP (2013). Integrative genomics viewer (IGV): high-performance genomics data visualization and exploration. Briefings in Bioinformatics.

[bib76] Villanueva RAM, Chen ZJ (2019). ggplot2: elegant graphics for data analysis (2nd ed.). Measurement.

[bib77] Wang Y, Li Y, Toth JI, Petroski MD, Zhang Z, Zhao JC (2014a). N6-methyladenosine modification destabilizes developmental regulators in embryonic stem cells. Nature Cell Biology.

[bib78] Wang X, Lu ZK, Gomez A, Hon GC, Yue YN, Han DL, Fu Y, Parisien M, Dai Q, Jia GF, Ren B, Pan T, He C (2014b). N6-methyladenosine-dependent regulation of messenger RNA stability. Nature.

[bib79] Wang Y, Li Y, Yue MH, Wang J, Kumar S, Wechsler-Reya RJ, Zhang ZL, Ogawa Y, Kellis M, Duester G, Zhao JC (2018). N6-methyladenosine RNA modification regulates embryonic neural stem cell self-renewal through histone modifications. Nature Neuroscience.

[bib80] Williams BR, Prabhu VR, Hunter KE, Glazier CM, Whittaker CA, Housman DE, Amon A (2008). Aneuploidy affects proliferation and spontaneous immortalization in mammalian cells. Science.

[bib81] Wu T, Hu E, Xu S, Chen M, Guo P, Dai Z, Feng T, Zhou L, Tang W, Zhan L, Fu X, Liu S, Bo X, Yu G (2021). clusterProfiler 4.0: a universal enrichment tool for interpreting omics data. Innovation.

[bib82] Xie W, Birchler JA (2012). Identification of inverse regulator-a (Inr-a) as synonymous with pre-mRNA cleavage complex II protein (Pcf11) in *Drosophila*. G3: Genes, Genomes, Genetics.

[bib83] Yan D, Perrimon N (2015). spenito is required for sex determination in *Drosophila melanogaster*. PNAS.

[bib84] Yang Y, Hsu PJ, Chen YS, Yang YG (2018). Dynamic transcriptomic m6A decoration: writers, erasers, readers and functions in RNA metabolism. Cell Research.

[bib85] Yang H, Shi X, Chen C, Hou J, Ji T, Cheng J, Birchler JA (2021). Predominantly inverse modulation of gene expression in genomically unbalanced disomic haploid maize. The Plant Cell.

[bib86] Yu GC, Wang LG, He QY (2015). ChIPseeker: an R/Bioconductor package for ChIP peak annotation, comparison and visualization. Bioinformatics.

[bib87] Zaccara S, Ries RJ, Jaffrey SR (2019). Reading, writing and erasing mRNA methylation. Nature Reviews. Molecular Cell Biology.

[bib88] Zhang S, Qi H, Huang C, Yuan L, Zhang L, Wang R, Tian Y, Sun L (2021a). Interaction of male specific lethal complex and genomic imbalance on global gene expression in *Drosophila*. Scientific Reports.

[bib89] Zhang S, Wang R, Huang C, Zhang L, Sun L (2021b). Modulation of global gene expression by aneuploidy and CNV of dosage sensitive regulatory genes. Genes.

[bib90] Zhang LD, Zhang S, Wang RX, Sun L (2022). Genome-wide identification of long noncoding RNA and their potential interactors in ISWI mutants. International Journal of Molecular Sciences.

[bib91] Zhang S, Wang R, Zhu X, Zhang L, Liu X, Sun L (2023). Characteristics and expression of lncRNA and transposable elements in *Drosophila* aneuploidy. iScience.

[bib92] Zhao BS, Wang X, Beadell AV, Lu Z, Shi H, Kuuspalu A, Ho RK, He C (2017). m(6)A-dependent maternal mRNA clearance facilitates zebrafish maternal-to-zygotic transition. Nature.

[bib93] Zhong SL, Li HY, Bodi Z, Button J, Vespa L, Herzog M, Fray RG (2008). MTA is an arabidopsis messenger RNA adenosine methylase and interacts with a homolog of a sex-specific splicing factor. The Plant Cell.

[bib94] Zhu C, Zhang S, Zhou C, Tian C, Shi B, Xu K, Huang L, Sun Y, Lin Y, Lai Z, Guo Y (2023). RNA methylome reveals the m(6)A-mediated regulation of flavor metabolites in tea leaves under solar-withering. Genomics, Proteomics & Bioinformatics.

