# Supplementary figures and images for: Dynamics and regulatory roles of RNA m6A methylation in unbalanced genomes
Source: eLife. 2025 Jan 24;13:RP100144. doi: 10.7554/eLife.100144 (PMC11759410; doi:10.7554/eLife.100144)

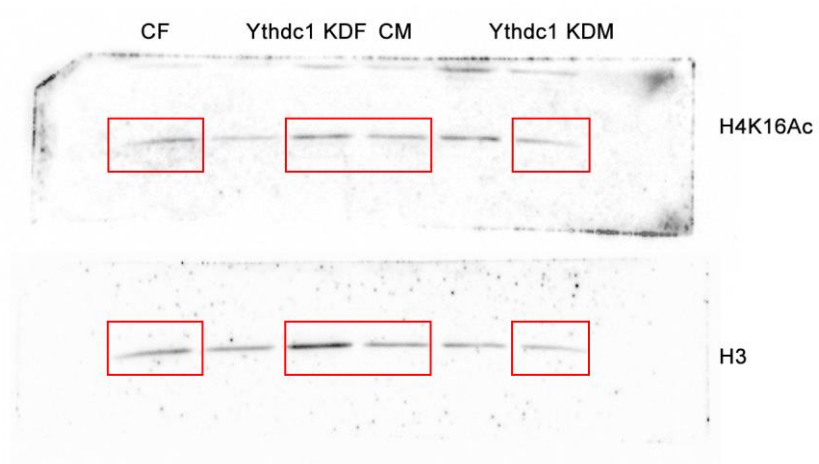

Figure 8—Source Data 1. Original membranes corresponding to Figure 8, panel D.

Supplement: Figure 8—source data 1. [file elife-100144-fig8-data1.zip › Figure 8—source data 1/Figure8_source data1.pdf]

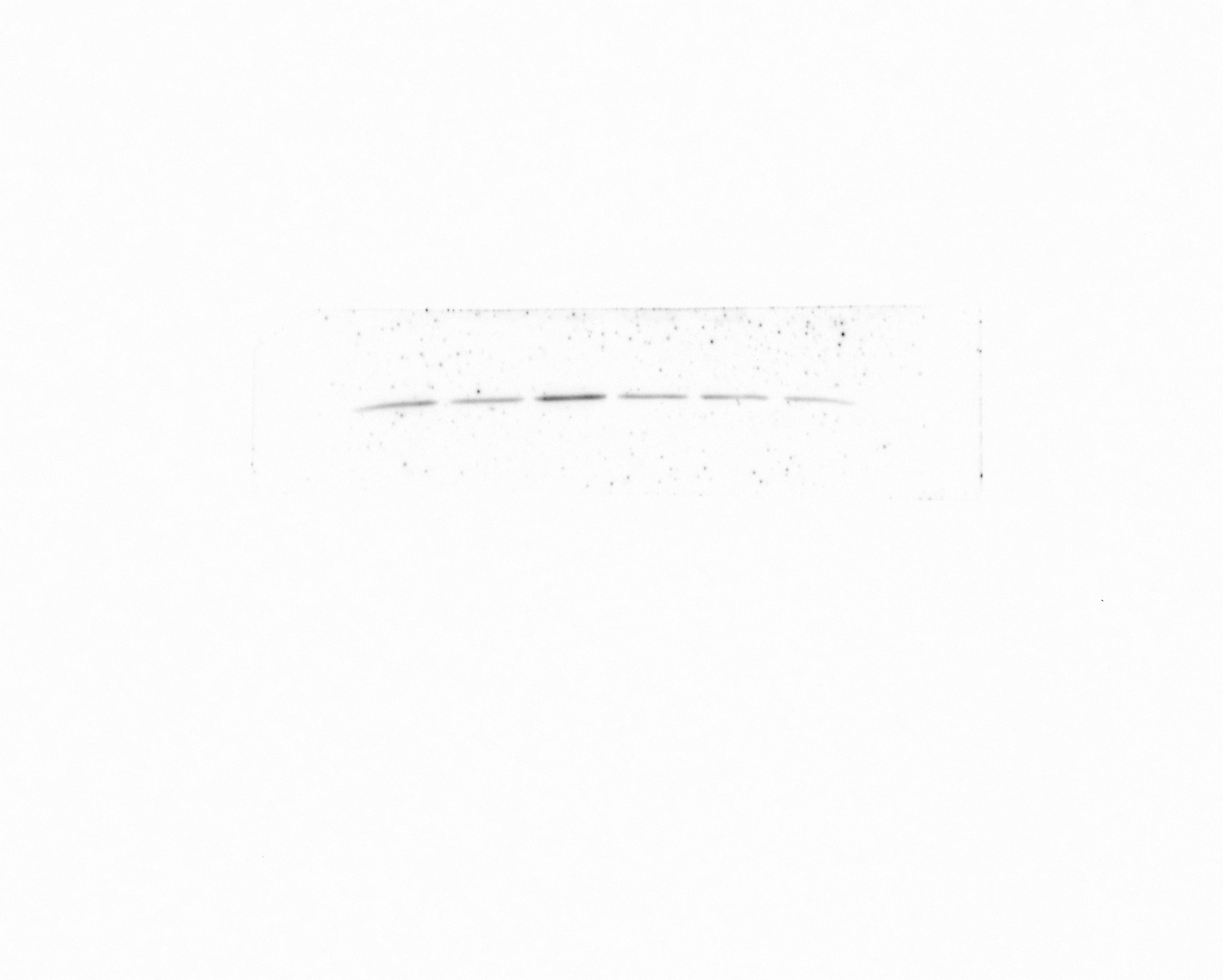

Supplement: Figure 8—source data 2. — Figure supplements. [file elife-100144-fig8-data2.zip › Figure 8—source data 2/H3.tif]

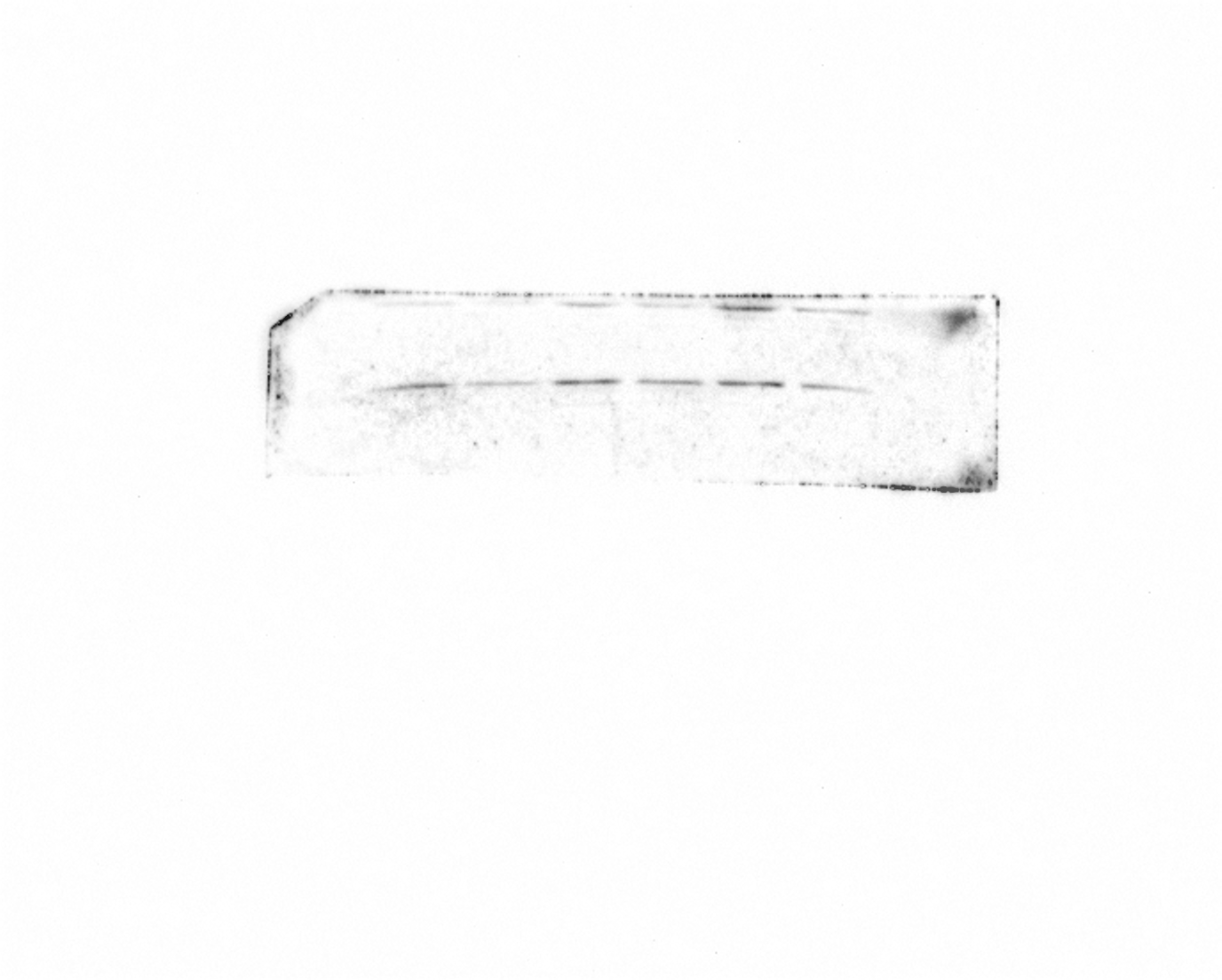

Supplement: Figure 8—source data 2. — Figure supplements. [file elife-100144-fig8-data2.zip › Figure 8—source data 2/H4K16Ac.tif]
